# Supplementary material for: Exploring negative emission potential of biochar to achieve carbon neutrality goal in China
Source: Nat Commun. 2024 Feb 5;15:1085. doi: 10.1038/s41467-024-45314-y (PMC10844326; doi:10.1038/s41467-024-45314-y)
Supplement: Supplementary file 1 — Supplementary Information [file 41467_2024_45314_MOESM1_ESM.pdf]

Supplementary Information

Exploring Negative Emission Potential of Biochar to Achieve Carbon Neutrality Goal in China

Xu Deng[1], Fei Teng[1]*, Minpeng Chen[2], Zhangliu Du[3], Bin Wang[4], Renqiang Li[5], Pan Wang[5]

[1] Institute of Energy, Environment and Economy, Tsinghua University, Beijing 100084, China
[2] School of Agricultural Economics and Rural Development, Renmin University of China, Beijing 100872, China
[3] College of Resources and Environmental Sciences, China Agricultural University, Beijing 100193, China
[4] Institute of Environment and Sustainable Development in Agriculture, Chinese Academy of Agricultural Sciences, Beijing 100081, China
[5] Key Laboratory of Ecosystem Network Observation and Modelling, Institute of Geographic Sciences and Natural Resources Research, Chinese Academy of Sciences, Beijing 100101, China
* Correspondence: tengfei@tsinghua.edu.cn

Contents:

Supplementary Note 1 Available biomass feedstocks 2
1.1 Agricultural residues 2
1.2 Forestry residues 3
1.3 Grass residues 3
1.4 Energy crops 4
1.5 Biomass abundance and soil texture 9
1.6 Available biomass feedstocks 10
Supplementary Note 2 Slow pyrolysis 12
Supplementary Note 3 Negative emission potential of biochar 19
Supplementary Note 4 Effects of biochar application on soil 19
4.1 Avoided soil greenhouse gas (GHG) emissions 19
4.2 Yield increasing 21
Supplementary Note 5 Economics of negative emission provided by biochar 22
Supplementary Note 6 Mitigation potential of biochar 24
Supplementary Note 7 Uncertainty analysis 25
Supplementary References 28

Supplementary Note 1   Available biomass feedstocks

1.1 Agricultural residues

The maximum theoretical potential represents the maximum amount of biomass feedstock available, assuming all biomass resources can be collected without competing uses. Theoretical agricultural residues ($Theo\_Fd_{agri}$, t/a) were calculated using formula (1-1), where Pro_k represents the production of crop k (k=1, 2, …, 16) in 2018 (t/a), r_k represents the residue-to-product ratio of crop *k*, which is shown in Supplementary Table 1.

$$Theo\_Fd_{agri} = \sum_{k=1}^{16} Pro_k \cdot r_k \quad (1\text{-}1)$$

Supplementary Table 1 Residue-to-product ratios of agricultural crops

Crops	Residues	Liu & Shen, 2007[1]	Bi et al., 2009[2]	Wang et al., 2013[3]	Cao et al., 2018[4]	Average
Rice	rice straw and	1	0.9	1.04	0.9	0.96
	rice hull		0.27	0.18		0.23
Wheat	wheat straw	1.1	1.1	1.28	1.17	1.16
Maize	maize straw	2	1.2	0.93	1.04	1.29
	maize cob		0.25	0.16		0.21
Other cereals	straw to seed		1.6	2.32		1.96
Soybean	soybean straw	1.7	1.6	1.35	1.6	1.56
Other beans	straw to seed		2			2.00
Tubers	cirrus to tubers	1	0.5	0.53	0.57	0.65
Peanut	peanut seedling and husk	1.5	1.11	1.26	1.14	1.25
Rapeseed	rapeseed straw	3	1.5	2.9		2.47
Sesame seed	sesame straw		2.2	1.89		2.05
Other oil crops	straw to seed	2	2.7	2.63		2.44
Cotton	cotton straw	3	9.2	3.34	3	4.64
Fiber	fiber straw		2.1	1.73		1.92
Sugar cane	sugarcane bagasse and leaf tip	0.1	0.3	0.5	0.3	0.30
Sugar beet	sugar beet bagasse, stem and leaf		0.18	0.42		0.30
Tobacco	by-product		1.6	0.66		1.13

The sustainable technical potential of available agricultural feedstocks (Sus_Fd_{agri}, t/a) implies that 95% of agricultural residues can be collected due to the loss during harvesting, processing, and transportation[5]. Then, feedstocks for basic use (BU) need to be deducted. In China, approximately 144 Mt/a of straw was used for feed, raw materials, and substrates feed according to the latest data[6]. Therefore, BU was assumed to be 144Mt/a.

$$Sus\_Fd_{agri} = \sum_{k=1}^{16} Pro_k \cdot r_k \cdot 95\%\text{-BU} \quad (1\text{-}2)$$

The current technical potential of available agricultural feedstocks (Cur_Fd_{agri}, t/a) represents the biomass resources available based on existing technologies and practices. According to current practice, the comprehensive utilization rate of straw in China is 88%, implying that at least 88% of crop residues can be collected currently[6]. Similarly, straw used for feed, raw materials, and substrates feed need to be deducted.

$$Cur\_Fd_{agri} = \sum_{k=1}^{16} Pro_k \cdot r_k \cdot 88\%\text{-BU} \quad (1\text{-}3)$$

1.2 Forestry residues

With reference to Fu et al.[7,8], 10 types of available forestry residues were considered: : 1) wood residues: wood nursery residues, forest pruning residues including residues of woody fruits and other pruning residues, wood logging residues, wood bucking residues, wood handling residues, firewood, and waste wood; 2) bamboo residues: bamboo pruning residues and waste bamboo; and 3) herbaceous fruit tree residues.

Forestry residues were calculated using the residue ratio of each type of wood, woody fruits or bamboo, the frequency of harvesting or pruning, and the corresponding area. In terms of waste wood and bamboo, calculations were based on the density of the bamboo and wood, the recycling rate of the waste product, and the products over 10 years ago. Detailed formulas can be seen in the study of Fu et al.[8]. Data on planted areas and production were obtained from 2018 Chinese Agricultural Yearbook[9], Forestry and Grassland Statistical Yearbook[10], and Ninth National Forest Resources Inventory at the province level[11]. Other coefficients were retrieved from the study of Fu et al.[12]

As a result, the maximum theoretical potential of forestry residues in each province j ($Theo\_Fd_{forest\,j}$, t/a) was calculated. The sustainable technical potential ($Sus\_Fd_{forest\,j}$) implies that 80% of forestry residues can be collected and utilized[13]. The current technical potential ($Cur\_Fd_{forest\,j}$, t/a) implies that 28% of forestry residues can be utilized[8].

Afterwards, available forestry residues were assigned to the 0.5° × 0.5° grid i based on land use type and gridded net primary productivity (NPP) in 2018. $Fd_{forest\,i}$ refers to the available forestry residues in each grid i (t/a), NPP_i refers to the NPP of 'woodland and other wooded land' in grid i (g/m²/a), and NPP_{ij} refers to the NPP of woodland and other wooded land in province j in which grid i is located (g/m²/a).

$$Fd_{forest\,i} = Fd_{forest\,j} \cdot \frac{NPP_i}{NPP_{ij}} \quad (1\text{-}4)$$

1.3 Grass residues

Total available grassland residues (Fd_{grass}, t/a) were calculated by using the statistical data as the total amount, and then were downscaled based on the NPP data as a weighting factor while excluding National Nature Reserve. First, according to the 2018 Annual Report on China's Forestry and Grassland Development[14], total fresh grass production on natural grasslands nationwide was reported as 1,099 Mt, which was equivalent to approximately 339 Mt of dry grass. Second, dry grass was assigned to each grid according to the formula (1-5). NPP_i refers to the NPP of grassland on grid i (g/m²/a), and NPP_{grass} refers to the aggregated NPP of grassland.

Then, grassland residues in National Nature Reserves were excluded. Finally, 288 Mt of grassland residues are available, which is considered as the maximum theoretical potential.

$$Theo\_Fd_{grass\ i}=Fd_{statistic}\cdot\frac{NPP_i}{NPP_{grass}} \tag{1-5}$$

The sustainable technical potential of available grassland residues implies collectable grass that is not used as feed. It is worth noting that China's livestock industry is still primarily based on grazing other than mowing, indicating that grassland residues are not available for biochar production in regions where the theoretical carrying capacity of grasslands is lower than the actual livestock load. To estimate available grassland residues, firstly, the carrying capacity of natural grasslands in China in 2018 – 26,717.12 million sheep units - was adopted from 2018 Annual Report on China's Forestry and Grassland Development[14]. Then, the carrying capacity were downscaled based on the NPP data, resulting in capacity on each grid ($loadcap_i$, sheep unit).

Secondly, we calculated the actual livestock load ($loadreal_i$, sheep unit). We obtained end-of-year livestock population data for cattle, horses, and sheep from the China animal husbandry and veterinary yearbook at 2018 level[15] and downscaled the statistical data using the Gridded Livestock of the World (GLW 4) 2015 livestock spatial data[16]. Then, based on Specification for Calculation of Grassland Livestock Carrying Capacity and Grass Livestock Balance[17], a yearly consumption of an adult sheep weighing 45 kg is 657 kg standard dry hay (*Cons,* t/sheep unit), and the conversion ratio of cattle (horse) to sheep is 5:1. Accordingly, we obtained the actual livestock load at the grid level. Finally, if the theoretical quantity was less than the actual quantity, the grassland residues available for biochar production were assumed to be zero. If the theoretical quantity exceeded the actual quantity, the difference was calculated as the available residue quantity, as shown in formula (1-6). It is worth noting that our calculations did not consider variations in utilization rates among different types of grasslands, potentially introducing bias to the results. Additionally, the utilization rate of grass for mowing might be higher, but we assumed that all grass for livestock comes from grazing, which might lead to underestimates.

$$Sus\_Fd_{grass\ i}=max\left(0,loadcap_i-loadreal_i\right)\cdot Cons \tag{1-6}$$

1.4 Energy crops

Marginal land is considered crucial for the production of second-generation bioenergy crops, specifically for lignocellulosic biomass crops. Certain energy crops can thrive in harsh environmental conditions. For example, sweet sorghum stands out as a prominent non-food energy crop in China for bioenergy production, owing to its high biomass yield, tolerance to drought and cold, and other remarkable attributes. Miscanthus is a perennial C4 plant with efficient photosynthetic pathways. Switchgrass is a perennial grass species capable of producing high biomass while thriving in low-quality soils and enduring extreme climatic conditions. Based on existing literature regarding the definition of marginal land (as shown in the Supplementary Table 2), in the maximum theoretical potential scenario, we defined marginal land as shrubland, the intertidal zone, bottomland, and unused land. In the sustainable technical potential scenario, marginal land was defined as shrubland and unused land, where shrubland in National Special Provision Shrubland Counties is excluded[18].

Supplementary Table 2 The definition of marginal land and results comparison between existing research and this study

Study	Marginal land	Area (Mhm^2)	Production	Energy crops
this study	Shrub land, the intertidal zone, bottomland, and unused land including sandy, Gobi, saline, marshland, bare land, and bare rocky land.	274.7	0.86 Gt/a	Miscanthus<br>Sweet sorghum<br>Switchgrass
	After considering climate conditions	53.8		
	Shrub land, and unused land including sandy, Gobi, saline, marshland, bare land, and bare rocky land, where the national special provision shrubland is excluded.	263.0	0.66 Gt/a	
	After considering climate conditions and soil qualities	50.5		
Zhang et al.,2020 [19]	Sparse grassland, Shrub land, Sparse Forest land, Moderate coverage grassland, High coverage grassland, Saline-alkali land, Bottomland, Bare land, Intertidal zone	184.9	1.76Gt/a<br>0.284Gt/a<br>9.7Mt/a	Miscanthus,<br>Switchgrass,<br>Jatropha seed
Nie et al., 2019 [5,20]	Tidal-flat land, sand land, saline-alkali soil land, swampland, bare land, etc.	91.49–102.09	12.30-20.46 EJ	Sweet sorghum
Jiang et al.,2019[21]	Shrub land, sparse forest land, grassland, shoal/bottomland, alkaline land and bare land.	49.65	13.57Mt	Sweet sorghum
Qin et al.,2018 [22]	Alkaline land, bare land, degraded land, waste land, and idle land	3 - 100		Review
Xue et al.,2016[23]	Sparse grassland, Shoal, Bottomland, Sand land, Gobi, Alkaline land, Wetland, Bare land, Bare rock	171.64(marginal land)<br>7.69(suitable)	0.135Gt/a<br>183.9TWh/a<br>0.212GtCO_2 eq/a	Miscanthus

Total production of energy crops (Fd_e, t/a) was calculated by multiplying the area of marginal land ($Area_m$, 1 km × 1 km each) and the yield of the highest yielding crop types (i.e., miscanthus, switchgrass, or sweet sorghum) in each plot of land. Energy crops can only be planted in areas with suitable environmental conditions, including precipitation, temperature and slope (Supplementary Table 3, Supplementary Figure 1), of which the data sources are presented in Supplementary Table 5. The yield at the provincial level was adopted from previous studies (Supplementary Table 4), as shown in formulas (1-7) and (1-8).

$$Fd_e = \sum_m Area_m \cdot max\{Yield\_mis_m, Yield\_swi_m, Yield\_swe_m\} \quad (1\text{-}7)$$

$$Yield_m = \begin{cases} Pt_m \cdot Yield_{province} & \text{inside regions with suitable environmental conditions} \\ 0 & \text{outside regions with suitable environmental conditions} \end{cases} \quad (1\text{-}8)$$

Besides, in terms of sustainable potential, since the soil quality would have influence on the growth potential of dedicated energy crops, we discounted the crop yield based on the soil quality data from Harmonized World Soil Database v1.2[24], which includes seven evaluation dimensions

such as nutrient availability, with each dimension having 1-7 levels. The description of soil quality said 'Only classes 1 to 4 are corresponding to an assessment of soil limitations for plant growth. Class 1 is generally rated between 80 and 100% of the growth potential, class 2 between 60 and 80%, class 3 between 40 and 60%, and class 4 less than 40%.' Therefore, we took the yield data from existing literature as the optimal yield, took the grades with lowest level for each grid, and discounted the productivity (Pt_m) according to the description. The resulting energy crop planting area is 50.5 Mhm^2, with a yield of 0.66 Gt/a, which is consistent with the range of existing estimates of 3-185 Mhm^2 [19,22,25] and of 13.57 Mt/a to over 1 Gt/a[19–21,23].

Supplementary Table 3 Conditions suitable for miscanthus, switchgrass, and sweet sorghum

Crops	Sources	AAT10[a]	slope	precipitation
Miscanthus	Zhang et al., 2021[26]	-	<25°	-
	Yan et al., 2021[27]	> 3,200℃	-	>800mm
	Value adopted in this study	> 3,200℃	<25°	>800mm
Switchgrass	Xue et al., 2016[23]	-	-	>400 mm
	Zhang et al., 2021[26]	-	-	381~762mm
	Yan et al., 2021[27]	> 2,500℃	<25°	>500 mm
	Value adopted in this study	> 2,500℃	<25	>500 mm
Sweet sorghum	Nie et al., 2019[20]	> 2,500℃	<25°	200~1,500 mm
	Jiang et al., 2019[21]	≥ 2,000℃	<25°	>400 mm
	Value adopted in this study	≥ 2,000℃	<25°	>400 mm

Note: a. AAT10 = Active accumulated temperature ≥10 ℃.

Supplementary Figure 1 (a)–(c) Regions with environmental conditions suitable for the cultivation of energy crops. The shapefile of national and provincial boundaries is quoted from the National Catalogue Service For Geographic Information, accessible at www.webmap.cn.

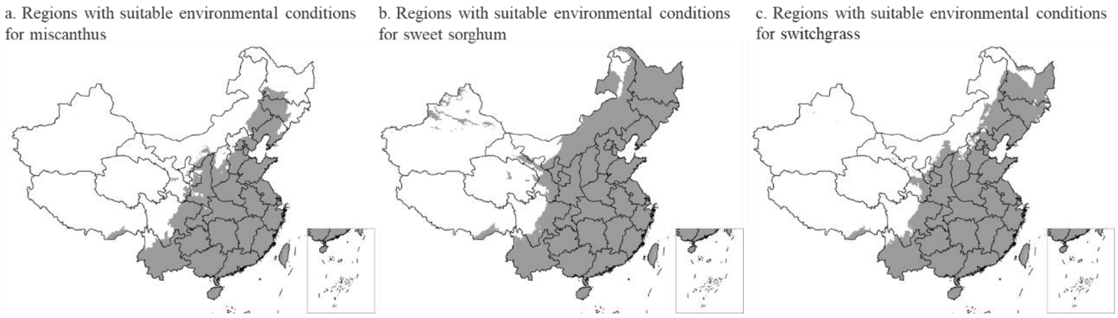

Supplementary Table 4 Yields of miscanthus, switchgrass, and sweet sorghum at the provincial level

Crops	Sources	Year	Model
Miscanthus	Zhang et al., 2020[19]	2017	MiscanFor
Switchgrass	Zhang et al., 2017[28]	2017	GEPIC
Sweet sorghum [a]	Nie et al., 2019[20]	2020	AquaCrop
DW t / (hm^2 a)	Miscanthus	Switchgrass	Sweet sorghum
Yunnan	22.9	8.9	14.7
Guangxi	23.5	11.7	11.8

Sichuan	13.2	8.2	14.9
Guizhou	18.7	9.8	13.3
Fujian	22.8	10	11.6
Inner Mongolia	6.6		13.6
Hunan	19.7	10	12.3
Shanxi	15.1		14.9
Hubei	19	10.3	12.7
Shaanxi	16.5	8.4	14.1
Jiangxi	21.5	10.2	12.2
Gansu	8.8		10.6
Guangdong	25.3	10.6	11.4
Heilongjiang	9.4	6.8	12.2
Xinjiang	3.5		9.3
Liaoning	15.5	7.2	13.9
Chongqing	18.5	9.5	13.9
Shandong	18.7	10.1	12.3
Hebei	13.2	7.3	13.6
Jilin	13.8		14.2
Anhui	20.3	12.2	12.6
Henan	18.1		10.9
Qinghai	3.5		2.8
Zhejiang	20.5	10	12.3
Ningxia	10.5		13.5
Hainan	26.6		10.9
Jiangsu	19.3	12.5	12.7
Beijing	16.4		14.1
Tibet	4.1		4.5
Tianjin	16.4		13.6
Shanghai			12.7

Note: a. Although sweet sorghum has high yield, apart from juice and sugar, only 50% of bagasse and pinnacles could be used for pyrolysis. 50% of the yields of sweet sorghum are presented in the table.

Supplementary Table 5 Sources of land use and climate data

Item	Resolution	Time period	Data sources
National Nature Reserve	Shape	2021 updated	Yunnan University
Land use	1 km	2018 retrieved	CNLUCC, 2018[29]
Temperature	27,830 m	2018-daily average	ERA5 [30]
Precipitation	1/24°	2018 retrieved	TerraClimate [31]
Slope	90m	2000 retrieved	SRTM data V4 [32]

Supplementary Figure 2 Marginal land and final planting area at the 1 km × 1 km level. In maximum theoretical scenario, marginal land refers to shrub land, the intertidal zone, bottomland,

and unused land including sandy, Gobi, saline, marshland, bare land, and bare rocky land (a). In the sustainable technical scenario, marginal land refers to shrub land, and unused land including sandy, Gobi, saline, marshland, bare land, and bare rocky land, where the national special provision shrubland is excluded (b). The land use type data are filtered from China's multi-period land use land cover remote sensing monitoring dataset[29]. Considering the climate constrains and nature reserves, final planting areas are shown in (c) and (d). The shapefile of national and provincial boundaries is quoted from the National Catalogue Service For Geographic Information, accessible at www.webmap.cn.

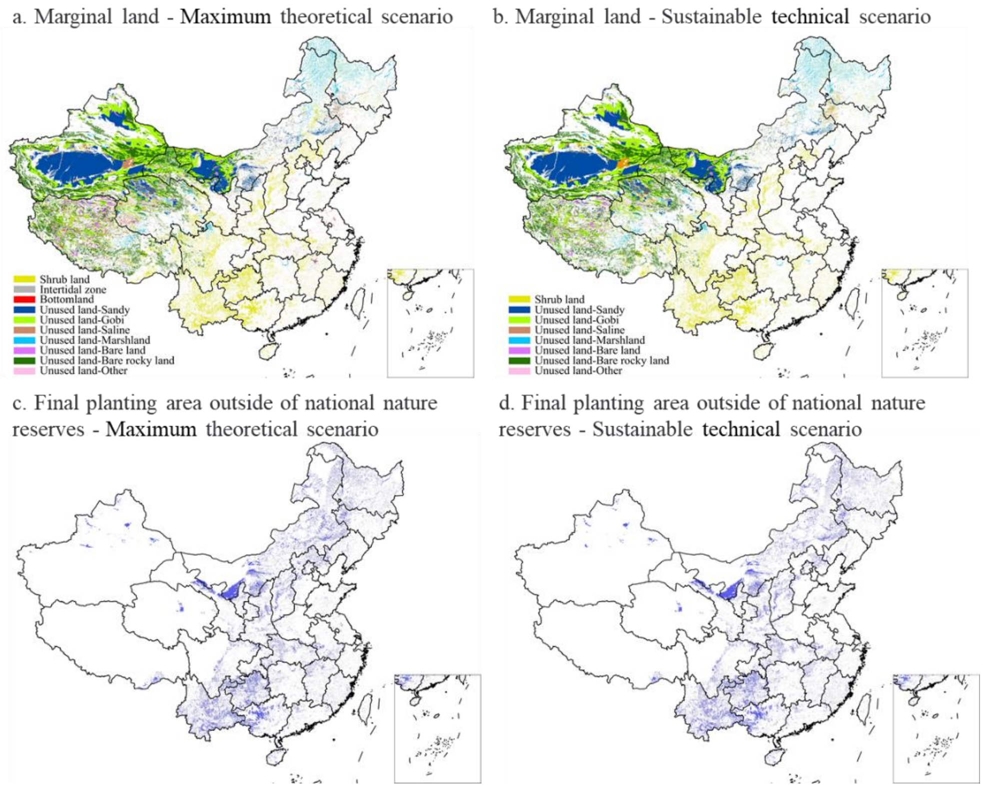

Supplementary Figure 3 (a, c) Yields and (b, d) types of potential energy crops on marginal land at the 1 km × 1 km level after best technology selection of sweet sorghum, switchgrass, and miscanthus. Note that switchgrass was not selected because of its relatively low yield. Although sweet sorghum has high yield, apart from juice and sugar, only 50% of bagasse and pinnacles could be used for pyrolysis. The shapefile of national and provincial boundaries is quoted from the National Catalogue Service For Geographic Information, accessible at www.webmap.cn.

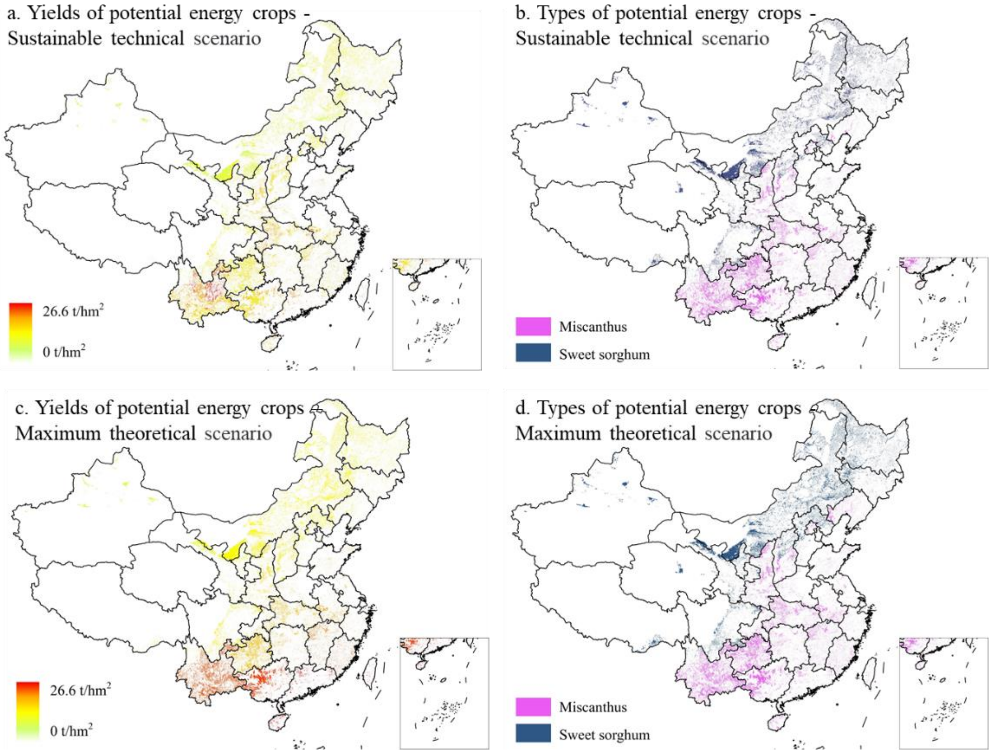

1.5 Biomass abundance and soil texture

Supplementary Figure 4 Biomass abundance. Notably, biomass abundance needs to achieve a lower limit, otherwise it will not only be difficult to harvest and transport, but also the investment might be uneconomic. According to our calculations, more than 80% of grids had more than 80,000 t of feedstock available. We set a lower limit of 80,000 t of feedstock in each 0.5° grid. If the feedstock of the grid was <80,000 t, the investment cost would be increased (by no more than 20%). Grids with low feedstock are mainly distributed in Southwest and Northwest China. The shapefile of national and provincial boundaries is quoted from the National Catalogue Service For Geographic Information, accessible at www.webmap.cn.

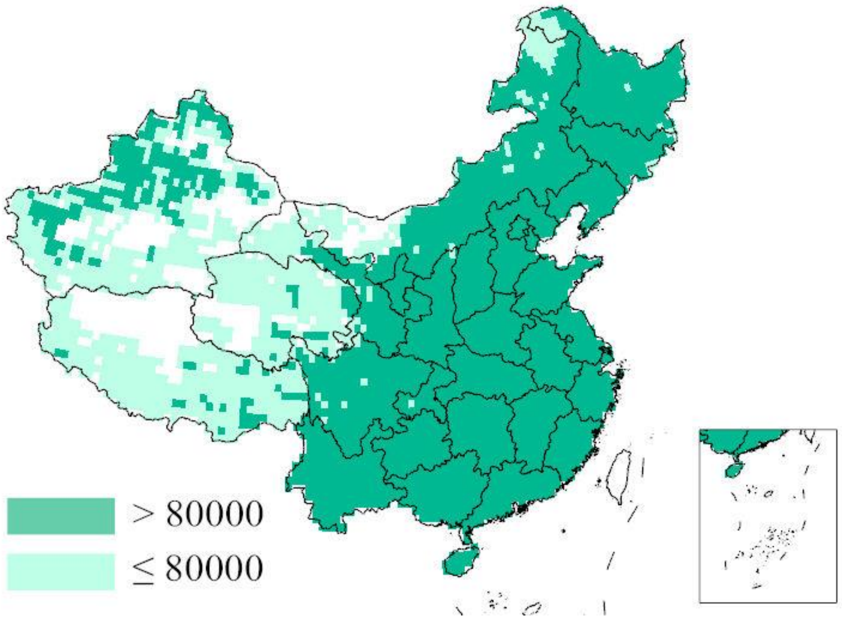

Supplementary Figure 5 Soil texture on a 0.5° × 0.5° grid. Resampling the data obtained from Harmonized World Soil Database (HWSD) [24]. Medium-textured soil consists of 6-silt, 7-silt loam, 8-sandy clay, 9-loam, and 10-sandy clay loam. Coarse-textured soil consists of 11-sandy loam, 12-loamy sand, and 13-sand. Fine-textured soil consists of 1-clays (heavy), 2-silty clay, 3-clay, 4-silty clay loam, and 5-clay loam. The shapefile of national and provincial boundaries is quoted from the National Catalogue Service For Geographic Information, accessible at www.webmap.cn.

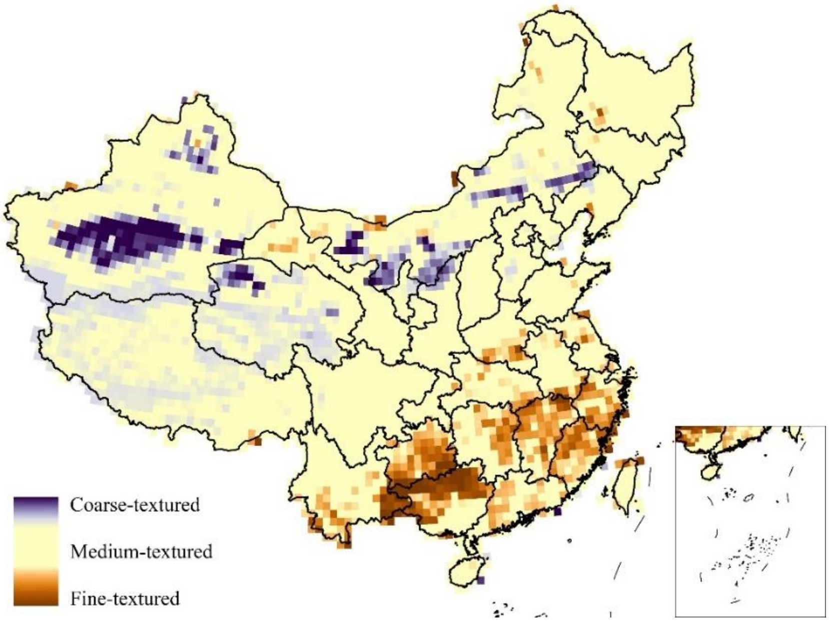

Supplementary Figure 6 PH on a 0.5° × 0.5° grid. Resampling the data obtained from Harmonized World Soil Database (HWSD) [24]. The shapefile of national and provincial boundaries is quoted from the National Catalogue Service For Geographic Information, accessible at www.webmap.cn.

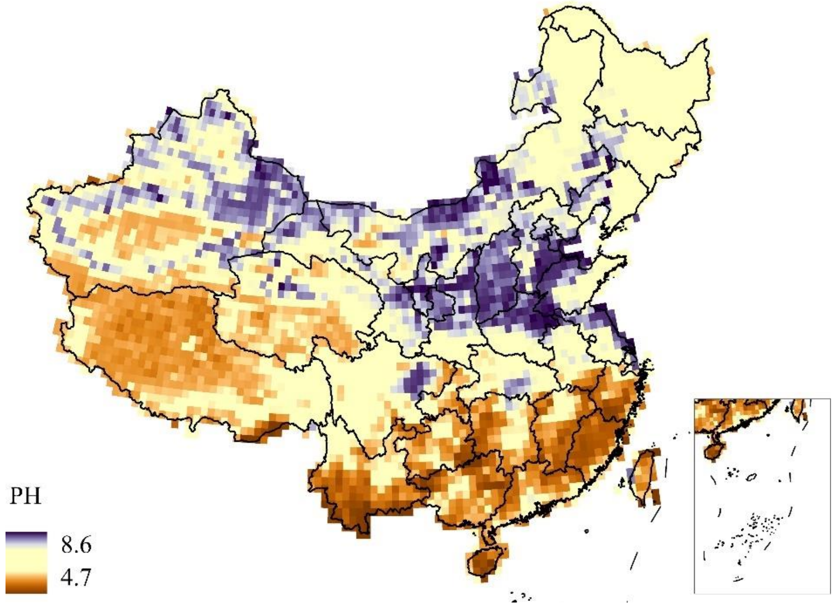

1.6 Available biomass feedstocks

Supplementary Figure 7 Types of biomass feedstock available for biochar production under the current technical scenario (in dry weight). a) Total biomass feedstock, including agricultural

residues and forestry residues. b) Agricultural residues that consist of 16 types of residues. c) Forestry residues that consist of 10 types of residues. Note that ‘forest pruning – other’ and ‘forest pruning - woody fruits’ both belong to forest pruning.

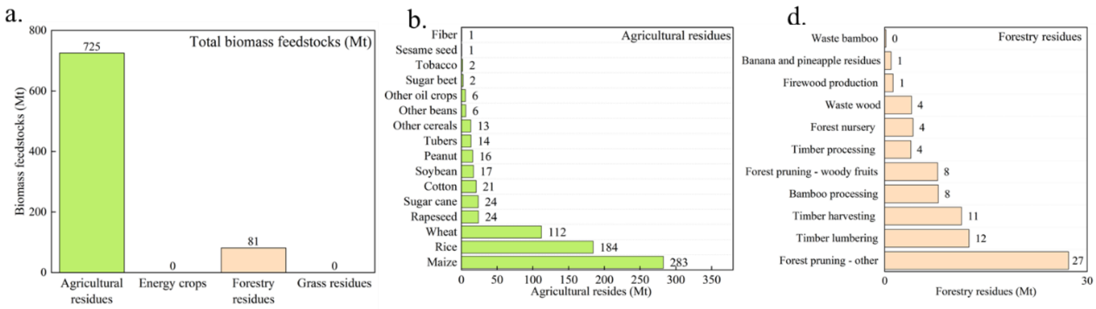

Supplementary Figure 8 Types of biomass feedstock available for biochar production under the sustainable scenario (in dry weight). a) Total biomass feedstock, including agricultural residues, energy crops, forestry residues, and grass residues. b) Energy crops that consist of sweet sorghum and miscanthus. Note that switchgrass is not listed owing to its low yield. c) Agricultural residues that consist of 16 types of residues. d) Forestry residues that consist of 10 types of residues. Note that ‘forest pruning - other’ and ‘forest pruning - woody fruits’ both belong to forest pruning.

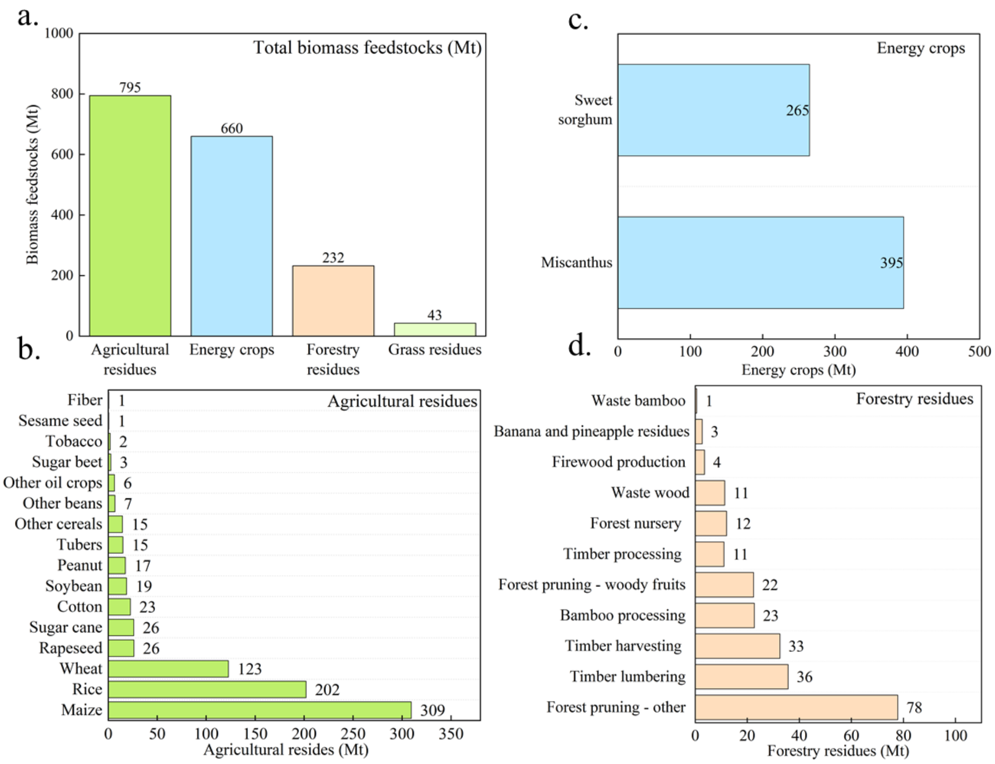

Supplementary Figure 9 Types of biomass feedstock available for biochar production under the maximum theoretical scenario (in dry weight). a) Total biomass feedstock, including agricultural residues, energy crops, forestry residues, and grass residues. b) Energy crops that consist of sweet sorghum and miscanthus. Note that switchgrass is not listed owing to its low yield. c) Agricultural residues that consist of 16 types of residues. d) Forestry residues that consist of 10

types of residues. Note that ‘forest pruning – other’ and ‘forest pruning – woody fruits’ both belong to forest pruning.

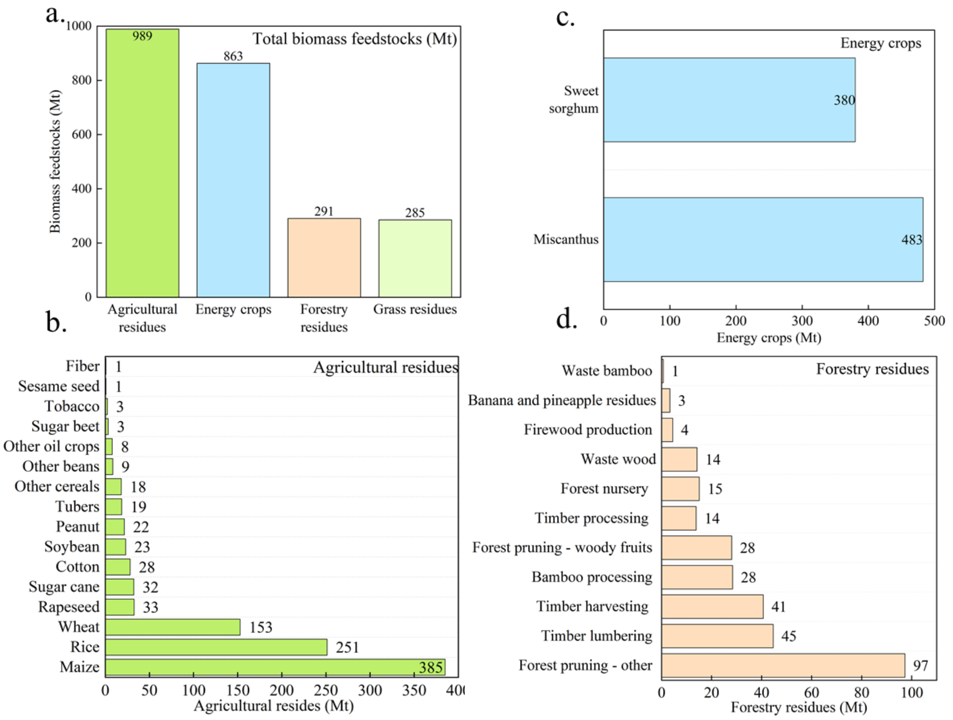

Supplementary Note 2 Slow pyrolysis

Supplementary Figure 10 Framework for Biochar Potential and Economics Evaluation. a) Scenarios setting of available biomass feedstocks. b) Properties of various types of biomass feedstocks, which serve as inputs for the empirical equations. c) Spatially explicit analysis of negative emission potential and economics of biochar. d) Determination of key parameters related to negative emissions, technology, and economics. The shapefile of national and provincial boundaries is quoted from the National Catalogue Service For Geographic Information, accessible at www.webmap.cn.

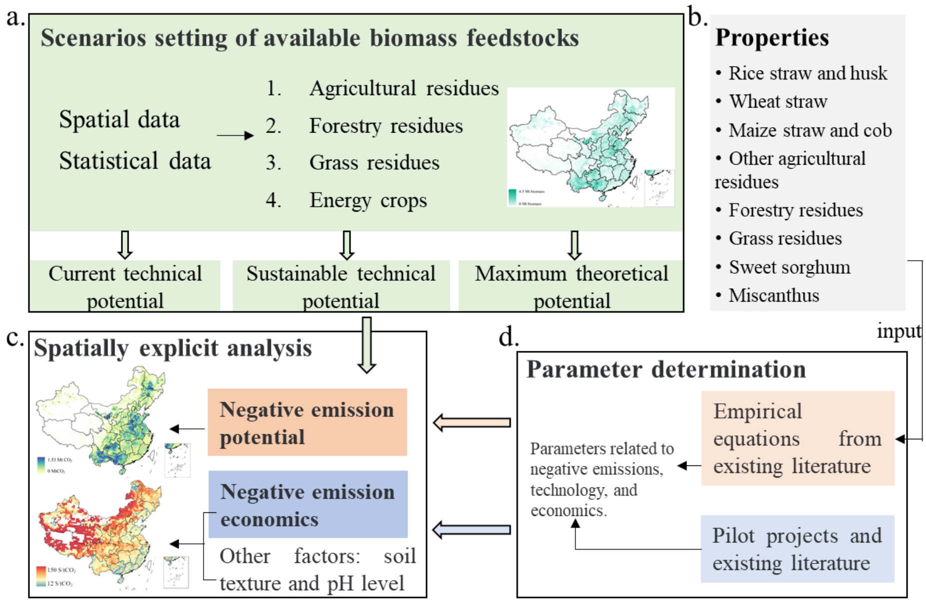

The outputs of slow pyrolysis vary considerably depending on biochar type and pyrolysis conditions such as temperature. To make a comprehensive assessment, it is not likely to directly adopt values from a single pilot project or experiment because existing pilot projects typically use only one or two types of biomass feedstock, and laboratory experiments on the properties of biochar often preclude studies of by-products.

We thus made efforts to process data from existing experiments or pilot projects with the aim of evaluating the negative emissions and economics of biochar produced from various biomass types, while maintaining conditions that were as consistent as possible. We tried to get comparable biochar parameters within a unified framework. Notably, the purpose of this data processing procedure was not to develop an accurate model of slow pyrolysis, but to make the outputs reported in the literature comparable. The steps were as follows.

First, we differentiated the weight conversion rate (α_i^{DAF}) and carbon content (β_i^{DAF}) of dry ash-free (DAF) biochar according to the GHG accounting framework of biochar application developed by Woolf et al.[33]. This framework is grounded in a comprehensive analysis of current empirical data. At the given temperature (T), the weight conversion rate of biochar[34] and carbon content[35] can be calculated with the input being the ash content of biochar (Bc_ash_i) and lignin content of biomass feedstocks (L_i). Then, we calculated the weight conversion rate (α_i) and the carbon content of resultant biochar (β_i). The formulas are shown as follows:

$$\beta_i = \beta_i^{DAF} \cdot (1 - Bc\_ash_i) \tag{2-1}$$

$$\beta_i^{DAF} = 0.93 - 0.92 \cdot e^{-0.0042 \cdot T} \tag{2-2}$$

$$Bc\_ash_i = Bm\_ash_i / \alpha_i \tag{2-3}$$

$$\alpha_i = \alpha_i^{DAF} + Bm\_ash_i \tag{2-4}$$

$$\alpha_i^{DAF} = 0.1261 + 0.5391 \cdot e^{-0.004 \cdot T} + 0.2733 \cdot L_i \tag{2-5}$$

The data of Bm_ash_i and L_i in term of the residues of main crops in China - rice husk, rice straw, wheat, maize straw, maize cob - were adopted from chemical composition analysis studies[36,37], which either research on typical Chinese varieties or averaged over multiple crop composition results. The data of Bm_ash_i and L_i of the grass residues and forestry residues were adopted from Woolf et al.[33], and those of miscanthus and sweet sorghum were adopted from Brosse et al.[38] and Lu et al.[39]. After calculation, α_i and β_i were shown in Supplementary Table 6.

Supplementary Table 6 Carbon contents and weight conversion rates of biochar produced from various feedstocks.

	Rice			Maize		Other			Energy crops	
Data	Straw	Husk	Wheat straw	Straw	Cob	agricultural residues	Grass residues	Forestry residues	miscanthus	Sweet sorghum
α_i^{DAF}	22.8%	25.2%	21.5%	22.3%	22.9%	23.4%	21.8%	25.3%	21.9%	24.2%
α_i	36.2%	42.9%	28.6%	28.6%	26.8%	29.2%	28.8%	27.5%	24.6%	25.0%
β_i	52.9%	49.3%	63.0%	65.2%	71.9%	67.2%	63.5%	77.2%	74.7%	81.0%
Bm_ash_i	13.4%	17.7%	7.1%	6.4%	3.8%	5.8%	7.0%	2.2%	2.7%	0.9%
L_i	15.6%	24.3%	10.7%	13.5%	15.9%	17.7%	11.8%	24.7%	12.3%	20.4%

Furthermore, to determine the high heating value (HHV_i) of biochar, we adopted empirical correlations based on proximate analysis that are developed by Qian et al.[40]. They selected ten kinds representative biomass to prepare biochar materials, and the correlation has been verified by experimental data and has generalizability.

$$HHV_i=-30.3\beta_i^{2} + 65.2Bc\_ash_i^{2} + 55.4\beta_i - 48.5Bc\_ash_i + 9.591 \tag{2-6}$$

Low heating value (LHV_i) of biochar is the heat of combustion reduced by the heat of water evaporation, which is calculated according to equation from US Environmental Protection Agency, as shown in formula (2-7), where W is weight percent of moisture, and H_i is weight percent of hydrogen. Then, using formula from Woolf et al.[34], hydrogen content of biochar can be calculated based on temperature, as shown in formula (2-8) and (2-9). Here, we simplified the calculation by assuming that W is 0.

$$LHV_i=HHV_i- 10.55\ (W + 9H_i) \tag{2-7}$$

$$H^{DAF}=-0.0041+0.1\cdot e^{-0.0024\cdot T} \tag{2-8}$$

$$H_i=H^{DAF}\cdot(1-Bc\_ash_i) \tag{2-9}$$

Then, LHV_i were calculated and shown in Supplementary Table 9.

Besides biochar, there are also by-products generated during the process of biomass pyrolysis. There are various technologies for biochar production, including carbonization, dry distillation, and gasification with multiple co-production techniques. The by-products may include syngas, bio-oil, wood vinegar, electricity, and others. Correspondingly, the energy efficiency varies significantly depending on the specific technology and process conditions.

To ensure that the identified technology type and the benefits from by-products align closely with reality in China, we both conducted literature research and obtained first-hand data from biochar production facilities, which aimed at facilitating a more accurate and reasonable economic evaluation.

Firstly, we selected the technology of biomass gasification for syngas and biochar co-production. Despite having a relatively lower weight conversion rate of biochar compared to other technologies, it has demonstrated significant economic benefits from both biochar and syngas, and is considered one of the key industrialization directions for biochar production in China. Domestic research teams, including Nanjing Agricultural University, Chinese Academy of Agricultural Engineering (Ministry of Agriculture), and Huazhong University of Science and Technology, have been actively involved in this field and performed a series of experiments, as shown in Supplementary Table 8, based on which, we have determined the energy efficiency, with a median value of 71% (μ, %).

Secondly, our survey on existing biochar pilot projects also supports the feasibility and development potential of biomass gasification for co-production technology, as shown in Supplementary Table 9. According to the practical data, we identified the types of by-products and their benefits. Given that in biomass gasification technology, gaseous tar and other products were almost combusted to powering the process, by-product benefits solely come from remaining usable syngas. Syngas has some main uses: for self-use to save heating expenses and conversion

into industrial steam or electricity. Here, syngas was assumed to be converted into industrial steam, as some of the pilot projects did (Supplementary Table 9). The heat value of medium-temperature and medium-pressure steam commonly used in industries at 400℃ and 4 MPa is approximately 3.278 GJ/t, with a price of 250 RMB per ton. The energy efficiency from syngas to steam is 92%. In this way, the benefits of syngas can be calculated.

Finally, to ensure energy balance, the energy preserved in syngas (Pro_gas_i, GJ/t biomass) was calculated by subtracting the energy preserved in the biochar from the total preserved energy in biomass and additional energy input (Ade_i, kWh/t biomass), as shown in formula (2-10). The system requires a few additional energy consumptions to sustain the operation, which is assumed to be 90 kWh/t biomass according to pilot projects (Supplementary Table 9). As a result, the benefits of the by-products and the production of various types of biochar would be differentiated. The data and data sources used are listed in Supplementary Table 7.

$$Pro\_gas_i=(Ade\cdot 0.0036\text{GJ/kWh}+Bio\_LHV_i)\cdot\mu-Bc\_LHV_i\cdot\alpha_i \quad (2\text{-}10)$$

Supplementary Table 7 Low heating value of biochar and biomass and energy preserved in syngas.

	Parameters diverse in feedstocks	Sources
Bc_LHV_i	low heating value of biochar (*GJ/t*)	Calculated based on proximate formula
Bio_LHV_i	low heating value of biomass (*GJ/t*)	Renewable Energy 2015 (China)[41]
Pro_gas_i	production of syngas (*GJ/t biomass*)	Calculated based on LHV_i of biochar, energy efficiency of pyrosis process

	**Rice**			**Maize**		**Other**			**Energy crops**	
**Data**	**Straw**	**Husk**	**Wheat straw**	**Straw**	**Cob**	**agricultural residues**	**Grass residues**	**Forestry residues**	**miscanthus**	**Sweet sorghum**
Bc_LHV_i	20.0	19.4	22.8	23.6	26.3	24.4	23.0	28.9	27.6	30.9
Bio_LHV_i	13.8	13.7	14.6	15.5	16.2	diverse	17.5	14.6	17.5	17.5
Pro_gas_i	2.79	1.64	4.09	4.47	4.71	diverse	6.03	2.68	5.85	4.92

Supplementary Table 8 Parameters of biomass gasification for syngas and biochar co-production technology.

No.	1	2		3	4	5	6			7	8
Technology Type	Fluidized bed gasification for co-generation of electricity, biochar and heat	Downdraft fixed bed biomass multi-production gasifier		Downdraft fixed bed biomass multi-production gasifier	Biomass Continuous Grading Pyrolysis	Carbon, gas and oil poly-generation pilot system	Straw carbonization polygeneration system			Mobile Equipment of Corn Stalk In-situ Returning Carbonization	Biomass intermediate pyrolysis poly-generation (BIPP) system
Team	Zhang et al., 2015[42] Nanjing Agricultural University	Cheng et al., 2015[43] Nanjing Agricultural University		Zhang et al., 2021[44] Nanjing Agricultural University	Zhao et al.,2016[45] Chinese Academy of Agricultural Engineering, Ministry of Agriculture	Cong et al.,2017[46] Chinese Academy of Agricultural Engineering, Ministry of Agriculture	Huo et al., 2017[47] Chinese Academy of Agricultural Engineering, Ministry of Agriculture			Zhao et al.,2023[48] Chinese Academy of Agricultural Sciences	Yang et al.,2021[49] Huazhong University of Science and Technology
Biomass Type	straw	furfural residues 20.87 MJ/kg	wasted mushroom inoculation bags 18.01 MJ/kg	apricot shell	corn stalk 16.82 MJ/kg	peanut husk	straw			corn stalk 15.19 MJ/kg 50 kg /h	14.7GJ/t
Temperature	600 ~ 800℃	600 ~ 800℃	400 ~ 600℃		550-600℃		450 ~ 500℃	450 ~ 500℃	450 ~ 500℃	600 ~ 800℃	550℃
Biochar Yield	15%-25%	30.0%	22.3%	25.9%	29. 97%	31.3%	260 ~ 300kg /t	280 ~ 320kg /t	280 ~ 320kg /t	21.0%	7.09GJ/t biomass
Biochar Calorific Value		26.18 MJ/kg	20.09 MJ/kg		26. 21 MJ/kg		20 ~ 23 MJ/kg	25 ~ 30 MJ/kg	25 ~ 30 MJ/kg	22 MJ/kg	25.68
Syngas Yield	1.7-2.3m^3/kg	2.49 m^3/kg	2.25 m^3/kg	1.87kg/kg	0.20m^3/kg	0.28m^3/kg	400m^3 /t	350m^3 /t	300m^3 /t	346 MJ/h	0.75GJ/t biomass
Syngas Calorific Value	1100-1200kcal/ Nm^3	4.86 MJ/m^3	4.92 MJ/m^3	1100~1300kcal/m^3		16.3MJ/m^3	3 ~ 6(MJ/ Nm^3)	10~12 (MJ/Nm^3)	8~10(MJ/Nm^3)		284.2
Bio-oil Yield	Gaseous tar is fed into the combustor for use in a				2.44%	3.40%	Small quantity, not intend	-	280 ~ 300kg /t biomass,		2.13GJ/t biomass

Bio-oil Calorific Value	gas-fired boiler, generating medium-temperature and medium-pressure steam to drive a steam turbine for power generation while also providing heat.						ed for production	-	5.9 MJ·kg -1		
Additional Energy Input for Equipment				17.71% accounts for heat loss, providing the heat required for activation reactions and the heat loss during the cooling process in the dry purification process	External heating with resistance wire 1.5kWh/kg biochar	Bio-oil, syngas	Spontaneous combustion	External heating	External heating	9MJ/h 10 kg biochar/h	14.7GJ/
Energy Efficiency	>80%			82%	53%	68%	52%	73%	80%	74. 6%	48.75% - 73.69%

Supplementary Table 9 Parameters of Pyrolysis Plants from Surveys

No.	1	2	3	4	5	6
Location	Shenyang	Inner Mongolia	Heilongjiang	Henan	Anhui	Guizhou
Commencement of Operation	2014	2021	2018	2016	2019	2018
Pyrolysis Technology	Fixed-bed biomass carbonization equipment, and co-production of syngas	Fixed-bed straw gasification, and co-production of biochar	Continuous anaerobic carbonization, and co-production of syngas and bio-oil	Gasification, and co-production of biochar	Gasification, and gas supply for 30t boiler co-production of biochar	Self-heating low-temperature pyrolysis
Biomass Type	Corn stalks, rice husks	Sunflower stalks	Corn stalks, hemp stalks	Peanut shells	Wood/chips, rice husk, Wood/chips	Tobacco stems, tobacco stalks, tobacco leaves, pine needles, etc.
Additional energy input	10kWh/t biomass	90kWh/t biomass	35kWh/t biomass	Liquefied gas ignition on start-up only	Diesel ignition on start-up only	22kWh/t biomass
Biochar Yield	1/5-1/4	1/5 Sunflower stalks<br>1/3 Maize cob	1/4	1/4	Rice husk 30%<br>Straw23%<br>Wood/chips 17%	1/3
Bio-oil Yield and Quantity	-	-	Utilized as raw material for asphalt, priced at 2000 RMB per ton.	-	-	-
Syngas Yield	Nodata	$100*10^6 m^2/a$<br>$3100KJ/Nm^3$	Nodata	Nodata	210000 t/a	Nodata

Syngas Utilization	For self-use, saving 100000 yuan per year on heating expenses	For self-use, drying products and providing heating in the factory area	For self-use, utilizing excess heat in the factory area	For self-use, using a rotary kiln dryer	Conversion to industrial steam at 250 RMB/ton	Continuing as a heat source for the pyrolysis equipment and converting excess heat to industrial saturated steam at 200 yuan/ton.
Other By-products	Nodata	For self-use, wood vinegar.	Nodata	For self-use, wood vinegar.	Nodata	Nodata

Supplementary Note 3 Negative emission potential of biochar

CO_2 fixed in one unit of feedstock from the atmosphere by photosynthesis, then transferred and permanently preserved in biochar Seq_i, (tCO_2/t biomass) can be calculated using the coefficients of α_i - weight conversion rate from feedstock i to biochar (%), β_i - carbon content of biochar produced by feedstock i (%), and per - the permanence rate of biochar over 100 years (%), as shown in formula (3-1). per (%) was calculated as 75.08%, based on the formula from Woolf et al.[33], as presented in formula (3-2) below, where T is the pyrosis temperature (550℃ in this study).

$$Seq_i = \alpha_i \cdot \beta_i \cdot \frac{44}{12} \cdot per \quad (3\text{-}1)$$

$$per = 0.28 + 8.56 \cdot 0.0001 \cdot T \quad (3\text{-}2)$$

The total negative emission potential of biochar (NE, tCO_2) was then calculated from Seq_i and Res_i (t biomass) - available feedstock biomass i, which includes various agricultural residues, grass residues, forestry residues, and potential energy crops:

$$NE = \sum_i Seq_i \cdot Res_i \quad (3\text{-}3)$$

Supplementary Note 4 Effects of biochar application on soil

4.1 Avoided soil greenhouse gas (GHG) emissions

The effects of avoidance of soil GHG emissions were calculated, assuming that biochar was returned to the locations from which the feedstocks were harvested. First, the optimal application rate γ was determined based on existing meta-analysis. Second, the avoided soil GHG emissions at an optimal biochar application rate were determined based on meta-analysis. Then, the actual biochar production was calculated from the feedstocks Res_i and weight conversion rate α_i. Finally, the total avoided soil GHG emissions ($ASGHG$, CO_2eq/a) were estimated by multiplying the actual biochar production by the effects of soil GHG emissions avoidance apportioned to one unit of biochar, as shown in formula (4-1):

$$ASGHG = \sum_i \left(Res_i \cdot \alpha_i \cdot \sum_j (f_{ij} \cdot E_j \cdot GWP100_j) \cdot \frac{1}{\gamma}\right) \quad (4\text{-}1)$$

where j represents soil CH_4, N_2O, and CO_2; f_{ij} is the rate of decrease in soil GHG emissions j after biochar application (%), which was adopted from meta-analysis and the diverse feedstocks i, as shown in Supplementary Table 10; E_j is the original soil emissions (t/ha/a). Here, $GWP100_{CH_4}$ was adopted as 28 and $GWP100_{N_2O}$ was adopted as 265.

Supplementary Table 10 Data and data sources related to soil GHGs

Parameters diverse in feedstocks		Sources	
f_{CH_4}	decreasing rate of soil CH_4 emissions (%)	Meta-analysis:	
f_{N_2O}	decreasing rate of soil N_2O emissions (%)	He et al., 2017[50]; Lee et al., 2021[51]; Orlova et al.,	
f_{CO_2}	decreasing rate of soil CO_2 emissions (%)	2019[52]; Song et al., 2016[53]	
E_{CH4}	original soil CH_4 emissions $(t \cdot ha^{-1} \cdot yr^{-1})$	0.2155	Tang et al., 2022[54]
E_{N2O}	original soil N_2O emissions $(t \cdot ha^{-1} \cdot yr^{-1})$	0.0025	Woolf et al., 2016[55]

E_{CO2}	original soil CO_2 emissions $(t \cdot ha^{-1} \cdot yr^{-1})$	0.3473	Wang et al., 2022[56]
γ	optimal application rate $(t \cdot ha^{-1})$	20t/ha or 2 t/ha [13,57]	

**Data**	**Rice**	**Wheat**	**Maize**	**Other agricultural residues**	**Grass residues**	**Forestry residues**	**miscanthus**	**Sweet sorghum**
f_{CH_4}	0.0%	0.0%	0.0%	0.0%	7.5%	-20.5%	8.0%	0.0%
f_{N_2O}	-20.0%	-18.0%	-18.0%	-18.0%	-2.5%	-40.4%	-33.0%	-18.0%
f_{CO_2}	12.0%	24.2%	20.7%	12.0%	17.1%	16.5%	17.1%	12.0%

Discussion on avoided soil greenhouse gas (GHG) emissions. Previous studies highlighted the important role of biochar in soil GHG avoidance. Biochar application is anticipated to potentially decrease N_2O emissions by around 20%, as indicated by previous experiments[53,58], assuming a general range of 10–30 t/hm^2 for the applied biochar amount. On the other hand, experiments have also shown that the continuous application of biochar at the rate of 2 t/hm^2/a could also lead to substantial avoidance of soil GHG emissions.

When the optimal application rate of biochar was assumed to be 20 t/hm^2, the avoided soil N_2O emissions were 3.84 Mt CO_2eq/a under the sustainable technical scenario, i.e., only 0.4% of the carbon captured by biochar. This is because the amount of biochar application is usually insufficient to achieve the optimal application rate in practice. Similarly, the avoidance of soil CH_4 emissions was limited. In croplands, the avoidance of CH_4 emissions was not considered because research on CH_4 is controversial and the results from meta-analysis were insignificant[50,53,59]. In other land, the effects can even be the opposite[50], which means that biochar application can increase soil CH_4 emissions . In terms of soil CO_2, based on meta-analysis[52,60], the priming effects in soil are also positive, which means that biochar can accelerate soil mineralization and increase soil CO_2 emissions. However, some studies have found that negative priming effects can be detected after a long period[61]. If the optimal application rate of biochar was assumed to be 2 t/hm^2, the avoided or increased GHG emissions would be 10 times greater than those under the assumption of an application rate of 20 t/hm^2. Further exploration on avoided soil greenhouse gas (GHG) emissions after applying biochar is needed in the future.

Supplementary Figure 11 Avoided GHG emissions with the assumption of an optimal biochar application rate of 20 t/hm^2.

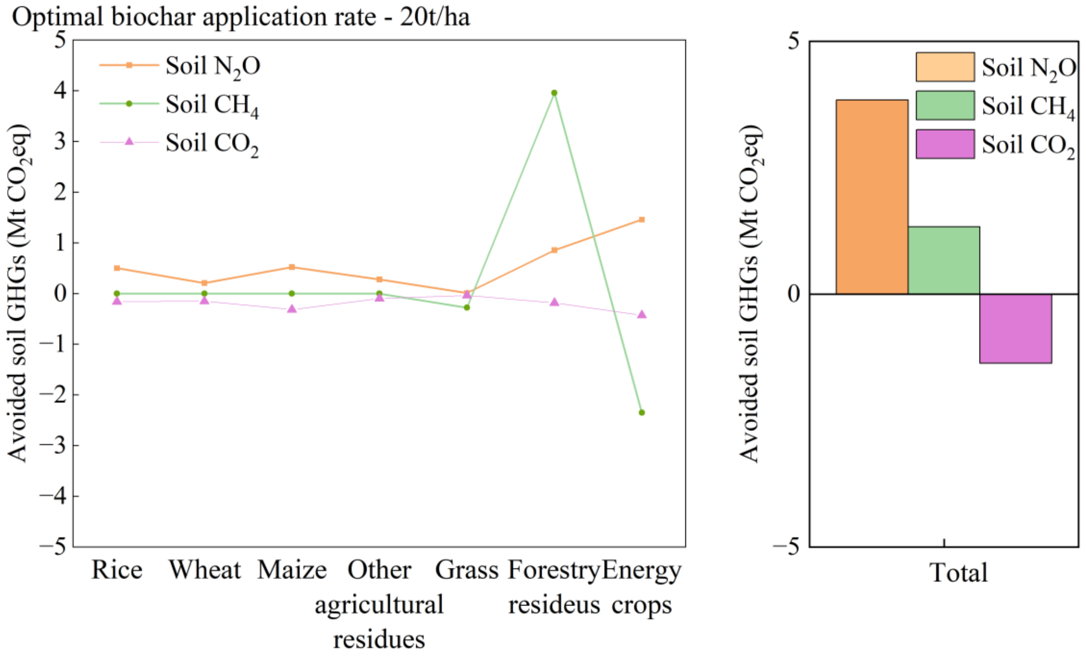

4.2 Yield increasing

The benefits of yield increasing from pyrolyzing one unit of biomass (B_yield_i) were calculated according to formula (4-2). The effects for one hectare of land at the optimal rate of biochar application were calculated by multiplying the yield ($Yield_i$, t/ha), price of crops (P_i, \$/t), and increasing rate of yield (θ_i, %). Then, the effects were apportioned per unit of biomass by dividing by the optimal biochar application rate γ and multiplying by the weight conversion rate α_i. Here, the value of γ was adopted as 20 t/ha for conservative estimation. The treatment applied here means to discount the effects of actual biochar application based on the optimal biochar application according to a linear relationship. This is a simplification and it can also be interpreted as a concentrated application on a small plot within a hectare of land after the biochar has been transported back to the harvesting site of the feedstock.

$$B\_yield_i=(\theta_i \cdot Yield_i \cdot P_i)\cdot\frac{1}{\gamma}\cdot\alpha_i \tag{4-2}$$

Supplementary Table 11 Data and data sources related to yield increase

**Parameters diverse in feedstocks**		**Sources**
$Yield_i$[1]	yield (t/ha)	China Statistical Yearbook 2019; Harvard Dataverse[62]
θ_i	increasing rate of yield (%)	Meta-analysis:<br>-Agricultural crops: Xia et al., 2023[63]; Yu et al., 2023[64];<br>-Other: Singh et al., 2022[57]
P_i	price of crops or economic forestry (\$/t)	State Administration of Grain, China

**Data**	**Rice**	**Wheat**	**Maize**	**Other agricultural crops**	**Grass**	**Forest**	**miscanthus**	**Sweet sorghum**
$Yield_i$	7.03	5.42	6.10	diverse	1.20	5.00	20.60	12.87
θ_i[2]	11.4%	14.4%	10.5%	diverse	53.0%	18.0%	45.1%	35.0%
P_i[3]	391	339	265	332	121	1662	175	175

Note: 1. Data of average yield was adopted from China Statistical Yearbook 2019. Data of yield at grid level was adopted from Harvard Dataverse[62] and our results about biomass spatial distribution; 2. The increasing rates of yield after biochar application on rice, wheat and maize are 9%, 14% and 13% according to Yu et al., 2023[64], and those are 14%, 15%, and 8% according to Xia et al., 2023[63]. For simplification, we adopted average value of these two latest studies. In terms of grass residues, forestry residues, and dedicated energy crops. We adopted the value from meta-analysis at the world level; 3. The price of energy crops is determined by multiplying 17.5GJ/t by 10$/GJ.

Given that biochar applied to soil with coarse or fine texture has been found to be more effective in improving crop yields, and soil pH also has an influence on the effectiveness of biochar application, we assumed that the effects of yield increase on coarse- and fine-texture soil or acidic soil would be 50% greater than the average value, while that on medium-texture soil or alkaline soil would be only half the average value[57,65], as shown in formula (4-3):

$$B\_yield_i' = \begin{cases} B\_yield_i \cdot (1\text{-}50\%), & \text{if biochar is applied to medium-texture soil or alkaline soil} \\ B\_yield_i \cdot (1\text{+}50\%), & \text{if biochar is applied to coarse- or fine-texture soil or acidic soil} \end{cases} \tag{4-3}$$

Supplementary Note 5 Economics of negative emission provided by biochar

We made the cost-benefit analysis (CBA) to identify the economics of negative emission provided by biochar. To be specific, the unit net cost of negative emissions (*UC*, $/t$CO_2$) was calculated using the net present value (*NPV*) and the total negative emissions (*TNE*) during the project lifetime (*T*). The biomass pyrolysis plant was assumed capable of processing 80,000 t of biomass per year over 20 years.

NPV of each plant can be formulated as:

$$NPV = -Inv + \sum_{t=1}^{T} \frac{(Income_t - Cost_t)\cdot(1+if)^t}{(1+r)^t} \tag{5-1}$$

$$UC = \frac{-NPV}{TNE} = \frac{-NPV}{Annually\ biomass\ pyrolysed \cdot lifetime \cdot Seq} \tag{5-2}$$

where $Income_t$ and $Cost_t$ are the income and cost in year *t*, respectively. For simplification, the discount rate *r* and inflation rate *if* is assumed to be the same in this study, implying these parameters doesn't change over time. Annual depreciation of the investment (Inv_t) is calculated based on *r* and *T*, as shown in formula (5-3)

$$Inv_t = \frac{Inv}{(1-1/(1+r)^T)/r} \tag{5-3}$$

Then, the net cost UC_i was calculated by dividing the costs and incomes of pyrolyzing one unit of biomass i by Seq_i:

$$UC_i = \frac{1}{Seq_i} \cdot (C\_pur + C\_bmstor + C\_inv + C\_o\&m + u\cdot k + u\cdot k\cdot \alpha_i + v\cdot \alpha_i - P\_gas\cdot Pro\_gas_i - B\_yield_i) \tag{5-4}$$

Where C_inv is the initial investment, C_pur is the purchasing cost, C_bmstor is the biomass

storage cost, $C\_o\&m$ is the operating and maintaining costs, u and k represent the cost of transporting biomass and biochar, where u represents the transport cost per ton of biomass or biochar, and k represents the distance between the purchasing source and pyrolysis plant, v is the cost of biochar application, and α_i is the conversion rate. The total income consists of syngas incomes, which were calculated using the price multiplied by the production amount, and the incomes from increased yields, B_yield_i. Relevant data are listed in Supplementary Table 12.

Supplementary Table 12 Data and data sources

**Common parameters**		**Data**		**Sources**
C_pur	purchasing cost ($/t biomass)	90 for energy crops and grass, 36 for other residues		Survey on pilot projects
C_bmstor	biomass storage cost ($/t biomass)	10		
C_inv	investment cost ($/t biomass)	4.8		
$C\_o\&m$	operating and maintaining cost ($/t biomass)	22.5		
P_gas	price of syngas ($/GJ)	10.6		
u	transport cost per ton biomass or biochar ($/t/km)	0.22		Research Report on Biomass Electricity Price Policy, 2018[66]
k^a	distance between the sources and pyrolysis plants (km)	21		Harvard Dataverse[62]
v	application cost of biochar ($/t biochar)	13.4		27.44$/ha, Woolf et al.,2016[55] 0.07$/kWh*53.11 kWh: 337.83 kg biochar, Yang et al.,2021[67]
Study	Technology type	*C_inv*	*C_o&m*	*C_pur*
Plant in Shenyang	Fixed-bed biomass carbonization equipment	-	-	36 -purchasing
Plant in Inner Mongolia	Fixed-bed straw gasification and co-production of biochar	4.1	17.9	42 -purchasing<br>6 - transport<br>4 - storage
Plant in Heilongjiang	Continuous anaerobic carbonization	5.7	13.5	33 -purchasing<br>5 - transport<br>5 - storage
Plant in Henan	Gasification and co-production of biochar	6.9	-	53- purchasing<br>15 - processing
Plant in Anhui	Gasification and gas supply for 30t boiler co-production of biochar	4.8	27.2	76<br>- purchasing, transport, storage, breaking
Plant in Guizhou	Self-heating low-temperature pyrolysis	6.1	42.0	33 - purchasing<br>8 - Transport

Note: a. The heating value of medium-temperature and medium-pressure steam commonly used in industries at 400 degrees Celsius and 4 MPa is approximately 3.278 GJ/t, with a price of 250 RMB per ton. The energy

efficiency of the combustion furnace is 92%. Based on the heat value, we have calculated the price of synthetic gas. b. Assuming that the plant is built at the center point of the grid, biomass will not be transported across the grids to control transportation costs.

Supplementary Note 6 Mitigation potential of biochar

Here, the mitigation potential of biochar included the carbon permanently preserved in biochar, the avoided soil GHG emissions, fossil fuel emission offsets, other life cycle emissions, and the avoided GHG emissions if residues are left in the field rather than used for biochar. The avoided soil GHG emissions were calculated as described in Note 4. Fossil fuel emission offsets refer to the emissions of fossil fuels avoided by using syngas. The emission factor for natural gas is 0.0543 tCO_2/GJ[41]. Emissions from electricity consumption during operation and maintenance was deducted (90KWh/t biomass). The grid emission factor is 0.581 tCO_2/MWh[68]. Additionally, we also accounted for other life-cycle emissions (125.4 $kgCO_2eq$/t biomass) during the feedstock processing and transportation, plant construction, water treatment, and biochar application[49,67,69,70]. In the context of biomass GHG emissions, only avoided CH_4 emissions were considered: rice residues left on paddy land have the potential to increase CH_4 emissions by 110.7%[71]. Finally, the mitigation potential that varies depending on the optimal biochar application rate was determined as 1,320–1,354 $MtCO_2eq$, i.e., 43% and 47% greater than the negative emission potential, respectively.

It is worth noting that the baseline used to assess the mitigation potential in this study corresponds to the current situation. Fossil fuel emission offsets, for example, was calculated as the differences between syngas and natural gas required to produce the same quantity of industrial stream. Another alternative option for energy production, namely BECCS, which is likely to be used in the future, is not considered in this study. The by-product of BECCS can be electricity, which implies a higher energy availability and is more ready-to-use than by-products of pyrolysis. On the other hand, limitations associated with BECCS are obvious, mainly including high carbon capture costs, poorly constructed carbon transport pipelines (especially in China where there is a serious mismatch of biomass sources and basin sinks), and the potential for carbon leakage and geological risks. Besides, the availability of residues for BECCS might be lower compared to biochar due to the necessity of maintaining soil fertility, and the left residues in field might also result in soil GHG emissions. In general, the comparison of biomass-based negative emission technologies needs further investigation.

Supplementary Figure 12 Mitigation potential of biochar under the sustainable technical scenario. The shaded bars represent the negative emission potential calculated in this study. All avoided GHG emissions represent mitigation potential. Note that avoided soil CO_2 can also be considered as negative emissions, but here we only count it as part of the mitigation potential.

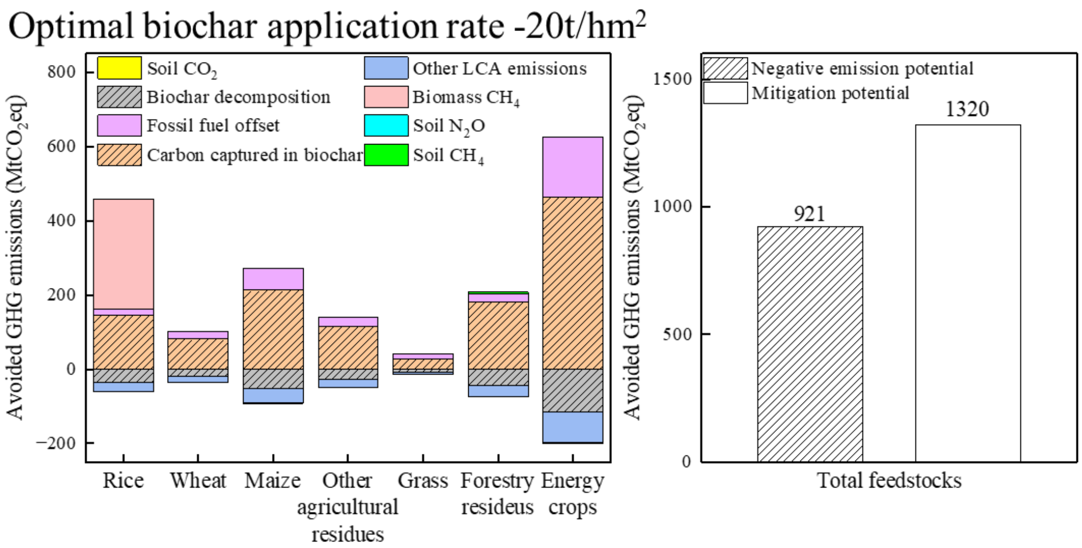

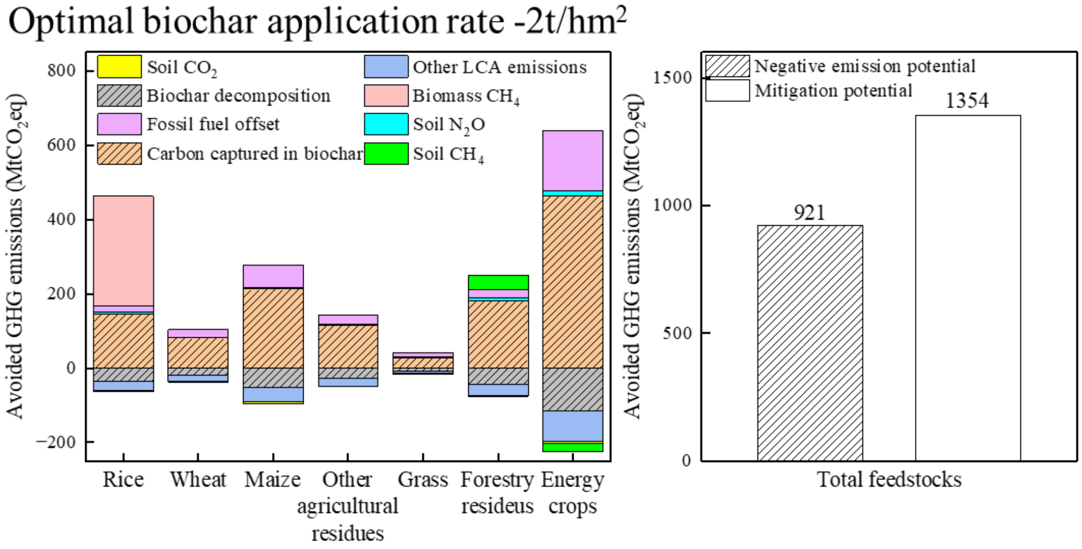

Supplementary Note 7 Uncertainty analysis

Using Monte Carlo simulation, we performed uncertainty analyses of the negative emission potentials and costs. The parameter ranges are shown in Supplementary Table 13. Random values were generated according to the normal distribution and triangular distribution. On the basis of existing literature, we adopted the mean μ and 95% confidence interval for each parameter, from which the standard deviation σ was calculated. After 10,000 iterations, we determined the uncertainty as a range. Consequently, the negative emission potential of biochar was estimated at 1.07 $GtCO_2$/a [0.68–1.46 $GtCO_2$/a, 95% confidence interval], and the negative emission cost of biochar was estimated at 92 \$/tCO_2 [-13–197 \$/tCO_2, 95% confidence interval].

Supplementary Table 13 Key parameters for uncertainty analyses

Triangular distribution	Lower limit	Mode	Upper limit
C_pur	30	36	90
C_inv	4.1	4.8	6.9
C_o&m	17.9	22.5	27.2
Biomass - rice [a]	-9%	-0.77%	5.6%

Biomass - wheat	-21%	-4.11%	13%
Biomass - maize	-29%	0.28%	41%
Biomass - forestry	11%	91%	192%
Biomass - energy crops	-4.3%	-0.63%	41.5%
θ_i	11%	18%	53%
Normal distribution	range	Mean (μ)	Standard deviation (σ)
u	0.15~0.25	0.20	0.0301
k	0~50	25	15.1989
v	10.7~16.1	13.4	1.6293
d	10%~40%	25%	0.0912
$Yield_i$	1.2~11.0	6.1	2.9668
P_i	265.3~398.0	331.6	40.3197
α_i	23.2%~34.8%	29%	0.0353
β_i	53%~79%	66%	0.0803
P_gas	8.5~12.7	10.6	1.2889
Energy efficiency	0.53~0.89	0.71	0.1079

Note a: detailed information is shown below.

When determining the range of available biomass resources, we assumed a close correlation between agricultural and forestry residues and their productions. To estimate future crop production, a common approach is to quantify the climate impact on crop yield and incorporate it into crop models or economic models to obtain crop production projections under various scenarios. According to existing studies on major grain crops, there is significant uncertainty in yield changes for major grain crops in the second half of the 21st century. For example, the change of rice yield could range from -9% to 5.6%, wheat from -21% to 13%, and maize from -29% to 41%[72–77]. Similarly, forestry residues are highly related to the forestry production. Studies have shown that under current climate conditions, carbon stocks are expected to significantly increase by 2060, and may even increase further under climate change scenarios like RCP4.5 and RCP8.5. Assuming linear growth, the increasing rate of biomass stock could range from 11% to 192% by 2060, with the median being 91%[78–80].

When conducting uncertainty analysis, we solely considered the influence of climate on biomass resources. However, it should be noted that socio-economic scenarios also have an impact on biomass production. According to AR6 Scenario Explorer and Database[81], IAMs that consider both socio-economic scenarios and climate scenarios show that the change of crop production compared to 2020 could range from -21% to 75.5% in 2060 (AIM/CGE, C3IAM, IMAGE), and the change of forestry production could range from -23% to -6 % in 2060 (AIM/CGE, IMAGE) in terms of ‘Counties of centrally-planned Asia; primarily China’. In general, the estimates of production are influenced by complex mechanisms, such as technological competition, carbon pricing, land-use policies, etc., which need to be explored in a broader framework.

As for energy crops, their productions are closely related to acreage and yields. Our assumptions about marginal land for energy plantations are conservative, and energy crop does not compete with food for land. To be specific, in the current technical scenario, the sustainable technical scenario and the maximum theoretical scenario of our study, we assumed no energy plantations, limited cultivation that adheres to ecological constraints, and all marginal land cultivation,

respectively. In terms of the impacts of future climate change on energy crop yields, we refer to Nie et al.[20] for an estimate of yield change intervals in 2060.

Supplementary Figure 13 Sensitivity analysis of parameters affecting the net costs of negative emissions

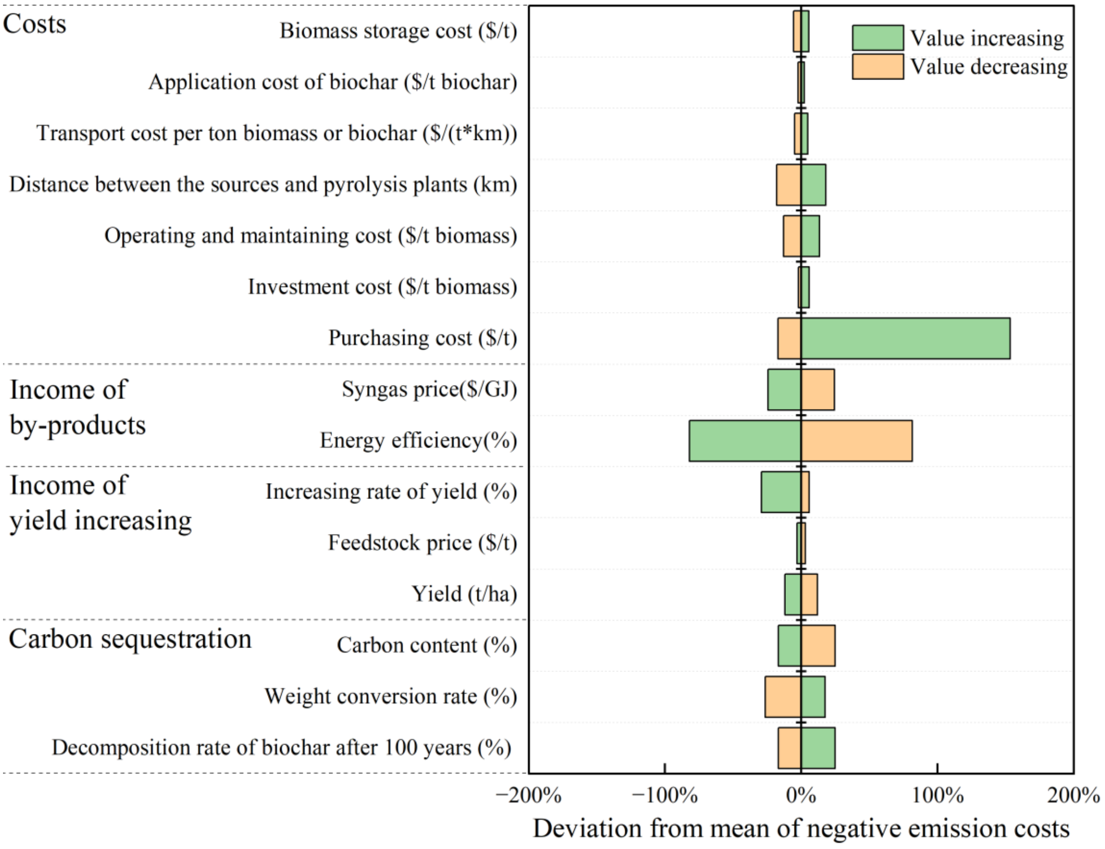

Supplementary Figure 14 Sensitivity analysis of parameters affecting the negative emission potentials.

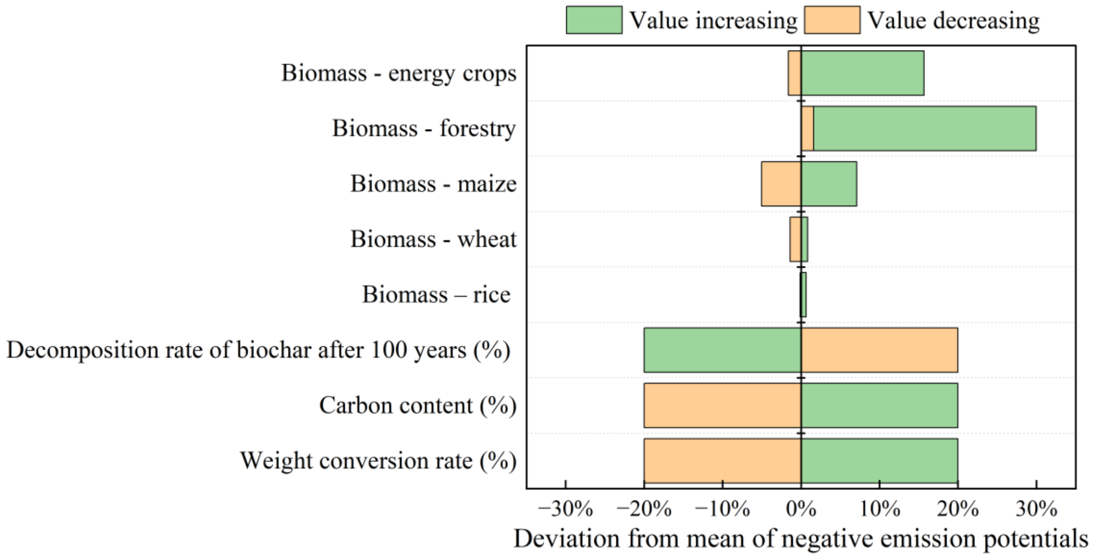

Supplementary References

1. Liu, G. & Shen, L. Quantitive appraisal of biomass energy and its geographical distribution in China. *Journal of Natural Resources* **22**, 9–19 (2007).
2. Bi, Y., Gao, C., Wang, Y. & Li, B. Estimation of straw resources in China. *Transactions of the CSAE* **12**, 211 − 217 (2009).
3. Wang, X. *et al.* Field crop residue estimate and availability for biofuel production in China. *Renewable and Sustainable Energy Reviews* **27**, 864–875 (2013).
4. Cao, Z., Huang, Y. & Hao, J. Multi-suitability comprehensive evaluation of crop straw resource utilization in China. *Research of Environmental Sciences* **1**, 179–186 (2018).
5. Nie, Y. *et al.* Spatial distribution of usable biomass feedstock and technical bioenergy potential in China. *GCB Bioenergy* **12**, 54–70 (2020).
6. Ministry of Agriculture and Rural Affairs of the People's Republic of China. *National report on the comprehensive utilization of crop straw* (Ministry of Agriculture and Rural Affairs of the People's Republic of China, Beijing, 2022).
7. Fu, T., Bao, W. & Xie, G. Methods for assessing resources of forestry residues. *Chinese Journal of Biotechnology* **34**, 1500–1509 (2018).
8. Fu, T., Ke, J. H., Zhou, S. & Xie, G. H. Estimation of the quantity and availability of forestry residue for bioenergy production in China. *Resources, Conservation and Recycling* **162**, 104993 (2020).
9. Ministry of Agriculture and Rural Affairs of the People's Republic of China. *2018 China Agricultural Yearbook* (China Academic Journal Electronic Publishing House, Beijing, 2019).
10. National Forestry and Grassland Administration. *2018 Forestry and Grassland Statistical Yearbook* (China Forestry Publishing House, Beijing, 2019).
11. National Forestry and Grassland Administration. *China Forest Resources Report 2014-2018* (China Forestry Publishing House, Beijing, 2019).
12. Fu, T., Wang, H. & Xie, G. Definition and assessment of coefficients for the calculation of forestry residues. *Chinese Journal of Biotechnology* **34**, 1693–1705 (2018).
13. Woolf, D., Amonette, J. E., Street-Perrott, F. A., Lehmann, J. & Joseph, S. Sustainable biochar to mitigate global climate change. *Nat Commun* **1**, 56 (2010).
14. National Forestry and Grassland Administration. *2018 Annual Report on China's Forestry and Grassland Development* (National Forestry and Grassland Administration, Beijing, 2020).
15. China Veterinary Animal Husbandry Yearbook Editorial Committee. *2019 China animal husbandry and veterinary yearbook* (China Agriculture Press, Beijing, 2019).
16. Gilbert, M. *et al*. *Gridded Livestock of the World - 2015 (GLW 4)*. Harvard Dataverse https://dataverse.harvard.edu/dataverse/glw_4 (2022).
17. The Quality and Technical Supervision Bureau of Sichuan Province. *Specification for Calculation of Grassland Livestock Carrying Capacity and Grass Livestock Balance* (The Quality and Technical Supervision Bureau of Sichuan Province, Sichuan, 2012).
18. National Forestry and Grassland Administration. *Circular on Clarifying the Boundaries of Forest Land Management and Regulating the Management of Forest Land on the Basis of the Results of the Third National Land Survey* (National Forestry and Grassland Administration, Beijing, 2023).
19. Zhang, B., Hastings, A., Clifton-Brown, J. C., Jiang, D. & Faaij, A. P. C. Modeled spatial

assessment of biomass productivity and technical potential of Miscanthus × giganteus, Panicum virgatum L., and Jatropha on marginal land in China. *GCB Bioenergy* **12**, 328–345 (2020).
20. Nie, Y. *et al.* Assessment of the potential and distribution of an energy crop at 1-km resolution from 2010 to 2100 in China – The case of sweet sorghum. *Applied Energy* **239**, 395–407 (2019).
21. Jiang, D., Hao, M., Fu, J., Liu, K. & Yan, X. Potential bioethanol production from sweet sorghum on marginal land in China. *Journal of Cleaner Production* **220**, 225–234 (2019).
22. Qin, Z. *et al.* Biomass and biofuels in China: Toward bioenergy resource potentials and their impacts on the environment. *Renewable and Sustainable Energy Reviews* **82**, 2387–2400 (2018).
23. Xue, S., Lewandowski, I., Wang, X. & Yi, Z. Assessment of the production potentials of Miscanthus on marginal land in China. *Renewable and Sustainable Energy Reviews* **54**, 932–943 (2016).
24. Fischer, G. *et al*. Global Agro-ecological Zones Assessment for Agriculture (GAEZ 2008). Harmonized World Soil Database v1.2 https://www.fao.org/soils-portal/data-hub/soil-maps-and-databases/harmonized-world-soil-database-v12/en/ (2008).
25. Qaseem, M. F. & Wu, A.-M. Marginal lands for bioenergy in China; an outlook in status, potential and management. *GCB Bioenergy* **13**, 21–44 (2021).
26. Zhang, A. *et al.* The implications for energy crops under China's climate change challenges. *Energy Economics* **96**, 105103 (2021).
27. Yan, D. *et al.* Are the planning targets of liquid biofuel development achievable in China under climate change? *Agricultural Systems* **186**, 102963 (2021).
28. Zhang, X., Fu, J., Lin, G., Jiang, D. & Yan, X. Switchgrass-Based Bioethanol Productivity and Potential Environmental Impact from Marginal Lands in China. *Energies* **10**, 260 (2017).
29. Xu, X. et al. China's multi-period land use land cover remote sensing monitoring dataset (CNLUCC). RESDC https://doi.org/10.12078/2018070201 (2018).
30. Copernicus Climate Change Service. *ERA5: Fifth generation of ECMWF atmospheric reanalyses of the global climate*. Climate Data Store https://cds.climate.copernicus.eu/cdsapp#!/home (2017).
31. Abatzoglou, J. T., Dobrowski, S. Z., Parks, S. A. & Hegewisch, K. C. Terraclimate, a high-resolution global dataset of monthly climate and climatic water balance from 1958-2015. *Scientific Data* **5**, 170191 (2018).
32. Jarvis, A., Reuter, H. I., Nelson, A. & Guevara, E. *Hole-filled SRTM for the globe Version 4*. CGIAR-CSI SRTM 90m Database https://srtm.csi.cgiar.org (2008).
33. Woolf, D. *et al.* Greenhouse Gas Inventory Model for Biochar Additions to Soil. *Environ. Sci. Technol.* **55**, 14795–14805 (2021).
34. Woolf, D., Lehmann, J., Fisher, E. M. & Angenent, L. T. Biofuels from Pyrolysis in Perspective: Trade-offs between Energy Yields and Soil-Carbon Additions. *Environ. Sci. Technol.* **48**, 6492–6499 (2014).
35. Neves, D., Thunman, H., Matos, A., Tarelho, L. & Gómez-Barea, A. Characterization and prediction of biomass pyrolysis products. *Progress in Energy and Combustion Science* **37**, 611–630 (2011).

36. Yao, X., Xu, K., Jia, Y. & Zhang, X. Thermogravimetric-Mass Spectrometry Analysis and Pyrolysis Kinetic of Rice Husk and Rice Straw. *Journal of Northeastern University(Natural Science) in Chinese* **37**, 426–430 (2016).
37. Zhao, J., Chen, J., Zhang, D. & Ghosh, S. Thermal stability and oxidation resistance of biochars derived from corn stalk and wheat stalk. *Journal of Agro-Environment Science* **38**, 458–465 (2019).
38. Brosse, N., Dufour, A., Meng, X., Sun, Q. & Ragauskas, A. Miscanthus: a fast-growing crop for biofuels and chemicals production. *Biofuels, Bioproducts and Biorefining* **6**, 580–598 (2012).
39. Lu, W., Zhang, Q., Zhou, H., Liu, C. & Cai, H. Study on thermal stabilities and mechanical properties of sweet sorghum slag/high density polyethylene composites. *Renewable Energy Resources (in Chinese)* **39**, 717–723 (2021).
40. Qian, C. *et al.* Prediction of higher heating values of biochar from proximate and ultimate analysis. *Fuel* **265**, 116925 (2020).
41. National Energy Administration. *China Renewable Energy Handbook 2015* (National Energy Administration, Beijing, 2015).
42. Zhang, Q. *et al.* Straw-type fluidized bed gasification for co-generation of electricity, biochar and heat. CN105368502A (2016).
43. Cheng, L. *et al.* Re-utilization of furfural residues and wasted mushroom inoculation bags by multi-production gasification technology. *Transactions of the Chinese Society of Agricultural Engineering (in Chinese)* **33**, 231–236 (2017).
44. Zhang, Y., Ma, H., Chen, D. & Zhou, J. Application case analysis of 3MW apricot shell gasification power generation co-production of activated carbon, heat and fertilizer. *Chemical Industry and Engineering Progress (in Chinese)* **40**, 1667–1674 (2021).
45. Zhao, L. *et al.* Equipment for Biomass Continuous Grading Pyrolysis. *Transactions of the Chinese Society for Agricultural Machinery (in Chinese)* **47**, 221–220 (2016).
46. Cong, H., Yao, Z., Zhao, L., Jia, J. & Lan, S. Development of carbon, gas and oil poly-generation pilot system based on biomass continuous pyrolysis. *Transactions of the Chinese Society of Agricultural Engineering (in Chinese)* **33**, 173–179 (2017).
47. Huo, L., Zhao, L., Yao, Z., Meng, H. & Cong, H. Utilization model and its efficiency analysis of biochar-gas-oil polygeneration by straw pyrolysis. *Transactions of the Chinese Society of Agricultural Engineering (in Chinese)* **33**, 227–232 (2017).
48. Zhao, L. *et al.* Mobile Equipment Study of Corn Stalk In-situ Returning Carbonization. *Transactions of the Chinese Society for Agricultural Machinery (in Chinese)* **54**, 357–363 (2023).
49. Yang, Q. *et al.* Prospective contributions of biomass pyrolysis to China's 2050 carbon reduction and renewable energy goals. *Nat Commun* **12**, 1698 (2021).
50. He, Y. *et al.* Effects of biochar application on soil greenhouse gas fluxes: a meta-analysis. *GCB Bioenergy* **9**, 743–755 (2017).
51. Lee, S.-I. *et al.* Biochar-induced reduction of N2O emission from East Asian soils under aerobic conditions: Review and data analysis. *Environmental Pollution* **291**, 118154 (2021).
52. Orlova, N., Abakumov, E., Orlova, E., Yakkonen, K. & Shahnazarova, V. Soil organic matter alteration under biochar amendment: study in the incubation experiment on the Podzol soils of the Leningrad region (Russia). *J Soils Sediments* **19**, 2708–2716 (2019).

53. Song, X., Pan, G., Zhang, C., Zhang, L. & Wang, H. Effects of biochar application on fluxes of three biogenic greenhouse gases: a meta-analysis. *Ecosystem Health and Sustainability* **2**, e01202 (2016).
54. Tang, Z., Zhang, J., Deng, A. & Zhang, W. Temporal-spatial characteristics and reduction approaches of methane emission from rice fields in China. *Chinese Journal of Eco-Agriculture* **30**, 582−591 (2022).
55. Woolf, D., Lehmann, J. & Lee, D. R. Optimal bioenergy power generation for climate change mitigation with or without carbon sequestration. *Nat Commun* **7**, 13160 (2016).
56. Wang, Y. *et al.* Effect of Mulching on Greenouse Gas Emissions from Paddy Field. *Crop Research* **36**, 1–8 (2022).
57. Singh, H., Northup, B. K., Rice, C. W. & Prasad, P. V. V. Biochar applications influence soil physical and chemical properties, microbial diversity, and crop productivity: a meta-analysis. *Biochar* **4**, 8 (2022).
58. Wang, X.-D. *et al.* Responses of greenhouse gas emissions to residue returning in China's croplands and influential factors: A meta-analysis. *Journal of Environmental Management* **289**, 112486 (2021).
59. Ji, C. *et al.* Variation in Soil Methane Release or Uptake Responses to Biochar Amendment: A Separate Meta-analysis. *Ecosystems* **21**, 1692–1705 (2018).
60. Rasul, M., Cho, J., Shin, H.-S. & Hur, J. Biochar-induced priming effects in soil via modifying the status of soil organic matter and microflora: A review. *Science of The Total Environment* **805**, 150304 (2022).
61. Lehmann, J. *et al.* Biochar in climate change mitigation. *Nat. Geosci.* **14**, 883–892 (2021).
62. International Food Policy Research Institute. Global Spatially-Disaggregated Crop Production Statistics Data for 2010 Version 2.0. Harvard Dataverse https://doi.org/10.7910/DVN/PRFF8V (2020).
63. Xia, L. *et al.* Integrated biochar solutions can achieve carbon-neutral staple crop production. *Nat Food* 1–11 (2023).
64. Yu, B. *et al.* Meta analysis on yield effect of biochar for staple crops in China. *Environmental Science (in Chinese)* **44**, 520–530 (2023).
65. Wang, Y., Villamil, M. B., Davidson, P. C. & Akdeniz, N. A quantitative understanding of the role of co-composted biochar in plant growth using meta-analysis. *Science of The Total Environment* **685**, 741–752 (2019).
66. Biomass Energy Industry Promotion Association. *Research Report on Biomass Electricity Price Policy* (Biomass Energy Industry Promotion Association, Beijing, 2018).
67. Yang, Q. *et al.* Country-level potential of carbon sequestration and environmental benefits by utilizing crop residues for biochar implementation. *Applied Energy* **282**, 116275 (2021).
68. Ministry of Ecology and Environment of the People's Republic of China. *Notice on Key Management Tasks for the 2022 Corporate Greenhouse Gas Emission Report* (Ministry of Ecology and Environment of the People's Republic of China, Beijing, 2022).
69. Yang, Q., Han, F., Chen, Y., Yang, H. & Chen, H. Greenhouse gas emissions of a biomass-based pyrolysis plant in China. *Renewable and Sustainable Energy Reviews* **53**, 1580–1590 (2016).
70. Clare, A. *et al.* Competing uses for China's straw: the economic and carbon abatement potential of biochar. *GCB Bioenergy* **7**, 1272–1282 (2015).

71. Liu, C., Lu, M., Cui, J., Li, B. & Fang, C. Effects of straw carbon input on carbon dynamics in agricultural soils: a meta-analysis. *Global Change Biology* **20**, 1366–1381 (2014).
72. Xie, W. *et al.* Climate change impacts on China's agriculture: The responses from market and trade. *China Economic Review* **62**, 101256 (2020).
73. Cui, Q., Ali, T., Xie, W., Huang, J. & Wang, J. The uncertainty of climate change impacts on China's agricultural economy based on an integrated assessment approach. *Mitig Adapt Strateg Glob Change* **27**, 25 (2022).
74. Wang, D. *et al.* Economic impacts of climate-induced crop yield changes: evidence from agri-food industries in six countries. *Climatic Change* **166**, 30 (2021).
75. Liu, B. *et al.* Similar estimates of temperature impacts on global wheat yield by three independent methods. *Nature Clim Change* **6**, 1130–1136 (2016).
76. Zhang, P., Zhang, J. & Chen, M. Economic impacts of climate change on agriculture: The importance of additional climatic variables other than temperature and precipitation. *Journal of Environmental Economics and Management* **83**, 8–31 (2017).
77. Zhao, C. *et al.* Temperature increase reduces global yields of major crops in four independent estimates. *Proceedings of the National Academy of Sciences* **114**, 9326–9331 (2017).
78. Jin, J. *et al.* Stand carbon storage and net primary production in China's subtropical secondary forests are predicted to increase by 2060. *Carbon Balance Manage* **17**, 6 (2022).
79. Dai, E., Wu, Z., Ge, Q., Xi, W. & Wang, X. Predicting the responses of forest distribution and aboveground biomass to climate change under RCP scenarios in southern China. *Global Change Biology* **22**, 3642–3661 (2016).
80. Zhou, L. *et al.* Carbon dynamics in woody biomass of forest ecosystem in China with forest management practices under future climate change and rising CO2 concentration. *Chin. Geogr. Sci.* **23**, 519–536 (2013).
81. Byers, E. *et al.* AR6 Scenarios Database. Zenodo https://zenodo.org/record/7197970 (2022).
