# Supplementary material for: Exploring negative emission potential of biochar to achieve carbon neutrality goal in China
Source: Nat Commun. 2024 Feb 5;15:1085. doi: 10.1038/s41467-024-45314-y (PMC10844326; doi:10.1038/s41467-024-45314-y)
Supplement: Supplementary file 3 — Reporting Summary [file 41467_2024_45314_MOESM3_ESM.pdf]

nature portfolio

Corresponding author(s): Fei TENG

Last updated by author(s): Dec 13, 2023

Reporting Summary

Nature Portfolio wishes to improve the reproducibility of the work that we publish. This form provides structure for consistency and transparency in reporting. For further information on Nature Portfolio policies, see our Editorial Policies and the Editorial Policy Checklist.

Statistics

For all statistical analyses, confirm that the following items are present in the figure legend, table legend, main text, or Methods section.

n/a	Confirmed	
☐	☒	The exact sample size (n) for each experimental group/condition, given as a discrete number and unit of measurement
☐	☒	A statement on whether measurements were taken from distinct samples or whether the same sample was measured repeatedly
☒	☐	The statistical test(s) used AND whether they are one- or two-sided<br>*Only common tests should be described solely by name; describe more complex techniques in the Methods section.*
☒	☐	A description of all covariates tested
☒	☐	A description of any assumptions or corrections, such as tests of normality and adjustment for multiple comparisons
☐	☒	A full description of the statistical parameters including central tendency (e.g. means) or other basic estimates (e.g. regression coefficient) AND variation (e.g. standard deviation) or associated estimates of uncertainty (e.g. confidence intervals)
☒	☐	For null hypothesis testing, the test statistic (e.g. F, t, r) with confidence intervals, effect sizes, degrees of freedom and P value noted<br>*Give P values as exact values whenever suitable.*
☒	☐	For Bayesian analysis, information on the choice of priors and Markov chain Monte Carlo settings
☒	☐	For hierarchical and complex designs, identification of the appropriate level for tests and full reporting of outcomes
☒	☐	Estimates of effect sizes (e.g. Cohen's d, Pearson's r), indicating how they were calculated

Our web collection on statistics for biologists contains articles on many of the points above.

Software and code

Policy information about availability of computer code

Data collection	Google Earth Engine
Data analysis	Matlab R2021a, Microsoft Excel 2019, Origin 2023b, Google Earth Engine, ArcGIS 10.6, https://github.com/DXDX97/Biochar_code_and_data

For manuscripts utilizing custom algorithms or software that are central to the research but not yet described in published literature, software must be made available to editors and reviewers. We strongly encourage code deposition in a community repository (e.g. GitHub). See the Nature Portfolio guidelines for submitting code & software for further information.

Data

Policy information about availability of data

All manuscripts must include a data availability statement. This statement should provide the following information, where applicable:

- Accession codes, unique identifiers, or web links for publicly available datasets
- A description of any restrictions on data availability
- For clinical datasets or third party data, please ensure that the statement adheres to our policy

Source data are provided with this paper, which have been deposited in Zenodo and GitHub [https://github.com/DXDX97/Biochar_code_and_data]. The data that support the main findings of this study are available in Supplementary Table 1-13. Raw data on crop spatial distribution, soil, land use type, and NPP used in this study are available in Harvard Dataverse 2010, Harmonized World Soil Database v1.2, RESDC and NASA, respectively. Other data are available from the corresponding author upon reasonable request.

Research involving human participants, their data, or biological material

Policy information about studies with human participants or human data. See also policy information about sex, gender (identity/presentation), and sexual orientation and race, ethnicity and racism.

Reporting on sex and gender	N/A
Reporting on race, ethnicity, or other socially relevant groupings	N/A
Population characteristics	N/A
Recruitment	N/A
Ethics oversight	N/A

Note that full information on the approval of the study protocol must also be provided in the manuscript.

Field-specific reporting

Please select the one below that is the best fit for your research. If you are not sure, read the appropriate sections before making your selection.

☐ Life sciences ☐ Behavioural & social sciences ☒ Ecological, evolutionary & environmental sciences

For a reference copy of the document with all sections, see nature.com/documents/nr-reporting-summary-flat.pdf

Ecological, evolutionary & environmental sciences study design

All studies must disclose on these points even when the disclosure is negative.

Study description	This study conducted a spatially explicit analysis to investigate the negative emission potential, economics, and priority deployment sites of biochar derived from multiple feedstocks in China. The data are quantitative.
Research sample	The sample is representative. This study calculated the biomass feedstock of 16 types of agricultural residues, 10 types of forestry residues, grass residues, and potential energy crops in China based on statistical and spatial data, which were then aggerated on a 0.5° × 0.5° grid. We used crop data from Harvard Dataverse 2010, soil data from Harmonized World Soil Database v1.2, land use data from Resource and Environment Science and Data Center (RESDC), and MODIS product MOD17A3HGF.006 from NASA. These data have high spatial resolution, meeting the requirements of the study.
Sampling strategy	We considered data from all provinces of the mainland, excluding data in national nature reserves to maintain the ecology. The data were computed and resampled to 0.5°x0.5° grids distributed across China. The sample size is sufficient since the study focuses on biochar potential and economics in China.
Data collection	Xu DENG used Google Earth Engine to collect data, and downloaded data from website of database.
Timing and spatial scale	Timing: Data was collected from 2021.10.12 to 2023.06.12.<br>Spatial scale: mainland China<br>-Crop data from Harvard Dataverse 2010: 10-km resolution<br>-Soil data from Harmonized World Soil Database v1.2: 30 arc-second raster<br>-Land use data from Resource and Environment Science and Data Center (RESDC): 1km*1km<br>-MODIS product MOD17A3HGF.006 from NASA: 500m*500m
Data exclusions	We assessed the biochar potential and economic viability in China, considering data from all provinces of the mainland. However, grid data within national nature reserves were excluded to adhere to the assumption of maintaining the ecology.
Reproducibility	Our data processing procedures are reproducibility. Data from existing databases, literature, and pilot projects were processed, filtered, and resampled according to this study's requirements.
Randomization	The main data analysis process did not include random group assignment, because we need to evaluate the potential and economic viability of biochar through all eligible grid data. We employed the Monte Carlo method for random sampling only in the uncertainty analysis, with n=10,000, and reported the mean and confidence intervals in the supplementary information.
Blinding	Blinding was not relevant to our study, since we need to evaluate the potential and economic viability of biochar through all eligible grid data.

Did the study involve field work? ☐ Yes ☒ No

Reporting for specific materials, systems and methods

We require information from authors about some types of materials, experimental systems and methods used in many studies. Here, indicate whether each material, system or method listed is relevant to your study. If you are not sure if a list item applies to your research, read the appropriate section before selecting a response.

Materials & experimental systems

n/a	Involved in the study
☒	☐ Antibodies
☒	☐ Eukaryotic cell lines
☒	☐ Palaeontology and archaeology
☒	☐ Animals and other organisms
☒	☐ Clinical data
☒	☐ Dual use research of concern
☒	☐ Plants

Methods

n/a	Involved in the study
☒	☐ ChIP-seq
☒	☐ Flow cytometry
☒	☐ MRI-based neuroimaging

Plants

Seed stocks	N/A
Novel plant genotypes	N/A
Authentication	N/A
